# Rare Chlorinated Fungal Metabolite and Alpha-Pyrones from an Endophytic Fungus Nigrospora sp.

**DOI:** 10.1021/acsomega.4c09190

**Published:** 2025-02-03

**Authors:** Chika C. Abba, Peter M. Eze, Sherif S. Ebada, Nchekwube K. Eze, Peter Proksch, Nicole Teusch, Festus B. C. Okoye, Chukwueweniwe J. Eboka

**Affiliations:** † Department of Pharmaceutical and Medicinal Chemistry, Nnamdi Azikiwe University, Awka 420261, Nigeria; ‡ School of Biological Sciences, Queen’s University Belfast, Northern Ireland, Belfast BT9 5DL, U.K.; § Department of Environmental Health Science, Nnamdi Azikiwe University, Awka 420261, Nigeria; ∥ Department of Pharmacognosy, Faculty of Pharmacy, Ain Shams University, Cairo 11566, Egypt; ⊥ Department of Pharmaceutical Microbiology and Biotechnology, Nnamdi Azikiwe University 420261, Awka, Nigeria; # Institute of Pharmaceutical Biology and Biotechnology, Heinrich Heine University, Düsseldorf 40225, Germany; ¶ Department of Pharmaceutical Chemistry, University of Benin, Benin City 1154, Nigeria

## Abstract

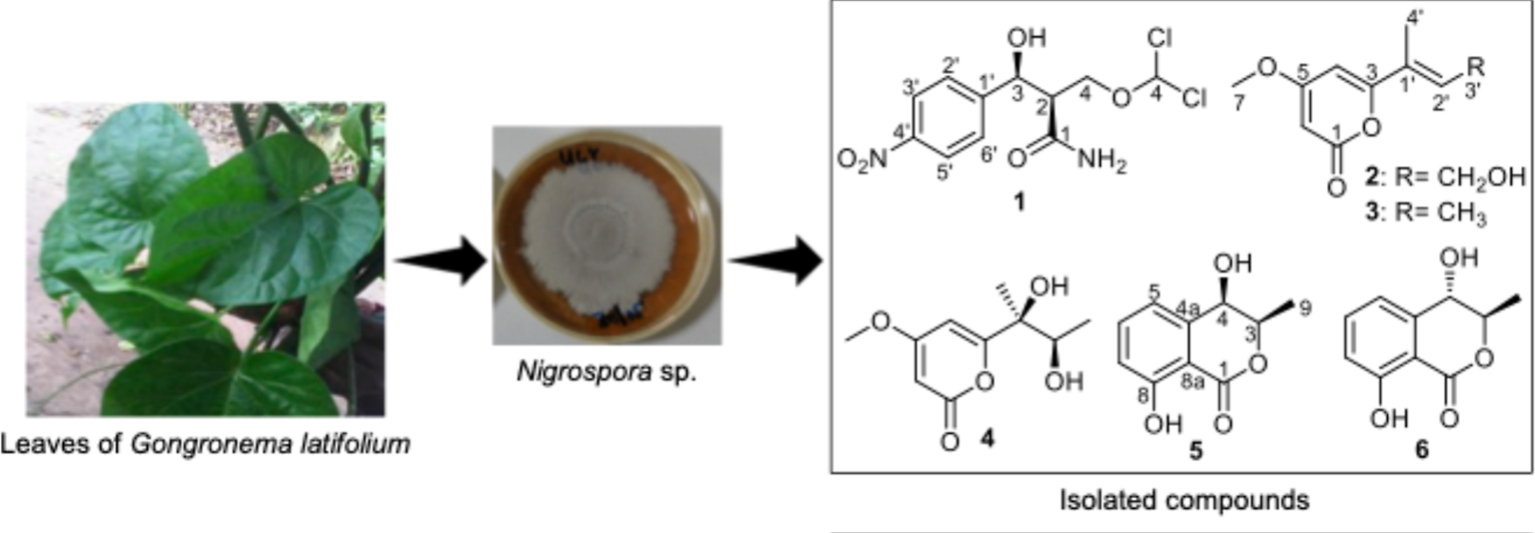

Endophytic microorganisms have been recognized as potential sources of new chemical entities with applications in the pharmaceutical, biotechnology, agricultural, and other industries. This study investigated the secondary metabolites produced by an endophytic Nigrospora sp. isolated from the Nigerian plant, *Gongronema latifolium*. Standard procedures were followed for fungal isolation, taxonomic identification, fermentation, and extraction of secondary metabolites. The antioxidant and antimicrobial properties of the fungal extract were assessed using the 1,1-diphenyl-2-picrylhydrazyl (DPPH) antioxidant assay and the agar-well diffusion assay, respectively. Various chromatographic and spectroscopic techniques were used to isolate, purify, and characterize compounds from the fungal extract. At 500 μg/mL, the fungal crude extract showed average antioxidant activity with a 48% inhibition. The extract also demonstrated moderate antimicrobial activity at 1 mg/mL against *Bacillus subtilis* and *Salmonella typhi*, with an inhibition zone diameter of 2 mm produced against both test strains. The fungal extract yielded six compounds, including the rare, chlorinated metabolite, acrodontiolamide (**1**), and five α-pyrone derivatives: hydroxypestalopyrone (**2**), pestalopyrone (**3**), pestalotiopyrone D (**4**), *cis*-4-hydroxymellein (**5**), and its *trans*-isomer (**6**). Interestingly, this is the second report of acrodontiolamide (**1**) in nature, after its first report in 1993. These compounds possess a wide range of known biological activities, including antimicrobial, antitumor, and cytotoxic effects, valorizing their potential in drug development.

## 1 Introduction

Many Nigerian plants have been investigated for their pharmacological properties, but far fewer have been studied for their endophytic populations. Endophytes, which are microorganisms living inside plants and producing secondary metabolites with specific functions, have been recognized as a valuable source for bioprospecting new biomolecules with potential benefits for mankind. Several studies have shown promising potential in endophytic microorganisms associated with Nigerian plants as sources of new bioactive chemicals for drug production and other applications.^1−11^ Interestingly, endophytes have been reported to produce beneficial bioactive natural products identical to compounds originally isolated from plants. Examples include paclitaxel, camptothecin, podophyllotoxin, hypericin, vinblastine, vincristine, and quercetin monoglycosides, with biological activities including antimicrobial, antiviral, antioxidant, anti-inflammatory, anticancer, immunomodulatory, and cardioprotective properties.^12−16^ This unique capability recognizes endophytes as ideal candidates for drug discovery research.

*Gongronema latifolium* is an edible tropical plant abundant in the rainforest zone of Nigeria. It grows as a climbing whose leaves are ethnomedicinally used in the treatment of various conditions, including worm infestation, stomach upset, diabetes, hypertension, malaria, and typhoid fever. Phytochemical and pharmacological studies on the plant revealed the presence of alkaloids, flavonoids, phenols, saponins, tannins, hydrocyanic acid, and phytic acid, which may be responsible for the various biological activities of the plant including antioxidant, antidiabetic, anti-inflammatory, and antimicrobial properties.^17−20^ Additionally, some phytocompounds with interesting bioactivities have been reported from the plant, including iloneoside (cytotoxic/anticancer), marsectohexol (α-amylase inhibitor/antidiabetic), and ajugoside (antioxidant).^21−23^

Several studies have documented interesting biomolecules from endophytic fungi associated with *G. latifolium*. Okoye and his fellow team isolated and identified bioactive compounds, including corynesidones A, C, and D, corynethers A and B, corynether lactone A, and their derivatives, from an endophytic fungus *Corynespora cassiicola* associated with the plant.^7,8^ These compounds have been reported to have interesting biological activities, including antimicrobial, cytotoxic, antioxidant, and aromatase inhibition.^24−26^ Additionally, Abba et al. reported the cytotoxic potential of secondary metabolites from several endophytic fungi of *G. latifolium*.^11^

Antibiotic-resistant pathogenic microbes have globally become a serious threat to the healthcare systems leading to immense human and economic losses. Therefore, the demand to explore new sources for novel antimicrobial agents is an urgent need.^27,28^ Fungal endophytes proved to be a prolific source of antimicrobial compounds against various pathogenic bacteria and fungi.^29^ On the other hand, metabolic reactions in the human body produces free radicals (reactive oxygen species, ROS) resulting in an increased oxidative stress and in turn damages the cellular components. These cellular damages can lead to various diseases such as cancers, neurodegenerative disorders, cardiovascular diseases and others.^30−32^ Therefore, there is a huge demand to attain novel antioxidant compounds combating the harmful effects of high oxidative stress yet to be of natural origin to minimize the adverse effects of synthetic antioxidants.^33−35^

In the course of our ongoing research targeting the identification of bioactive molecules from endophytic fungi, particularly those with antimicrobial and/or antioxidant properties, we explored the total organic extract of the endophytic fungus Nigrospora sp. derived from the Nigerian medicinal plant *G. latifolium* Benth. (Apocynaceae) and characterized its secondary metabolites. The fermentation extract was selected based on its results, which revealed moderately active antimicrobial and antioxidant properties. Further chemical investigation of the fungal extract revealed a rare chlorinated fungal metabolite and five α-pyrone derivatives.

## 2 Results and Discussion

### 2.1 Fungal Identification

This study reports the isolation of an endophytic fungus from the leaves of *G. latifolium* growing in southeastern Nigeria. Following taxonomic identification through DNA amplification and sequencing of its ITS regions, the fungus was identified as Nigrospora sp. The obtained nucleotide sequence was deposited in GenBank under the accession number MF327715 (Table 2). This is the first report of the isolation of endophytic Nigrospora sp. from *G. latifolium*. There is no indication that this fungus is pathogenic to the plant, as it was isolated from the internal tissues of healthy *G. latifolium* leaves using standard procedures for endophytic fungi isolation.

**Table 1:** Primers Used for PCR^68^

gene name	primer sequences (5′→ 3′)	direction
ITS1	TCCGTAGGTGAACCTGCGG	forward
ITS4	TCCTCCGCTTATTGATATGC	reverse

**Table 2:** Identification of the Endophytic Fungus, Determined by Amplifying the ITS1 and ITS2 Regions of Its rDNA

organism	host	primer	nucleotide Sequence (FASTA format)	accession number^a^
Nigrospora sp.	Gongronema latifolium	ITS1/ITS4	CTACCCGGGACCCCGCGCCCCGGGCGGCCCGCCGGCGGACAAACCAAACTCTGTTATCTTCGTTGATTATCTGAGCGTCTTATTTAATAAGTCAAAACTTTCAACAACGGATCTCTTGGTTCTGGCATCGATGAAGAACGCAGCGAAATGCGATAAGTAATGTGAATTGCAGAATTCAGTGAATCATCGAATCTTTGAACGCACATTGCGCCCATTAGTATTCTAGTGGGCATGCCTGTTCGAGCGTCATTTCAACCCCTAAGCACAGCTTATTGTTGGGCGTCTACTCCTGTAGTGCCTCAAAGACATTGGCGGAGCGGCAGTAGTCCTCTGAGCGTAGTAATTCTTTATCTCGCTCTACCCGGGACCCCGCGCCCCGGGCGGCCCGCCGGCGGACAAACCAAACTCTGTTATCTTCGTTGATTATCTGAGCGTCTTATTTAATAAGTCAAAACTTTCAACAACGGATCTCTTGGTTCTGGCATCGATGAAGAACGCAGCGAAATGCGATAAGTAATGTGAATTGCAGAATTCAGTGAATCATCGAATCTTTGAACGCACATTGCGCCCATTAGTATTCTAGTGGGCATGCCTGTTCGAGCGTCATTTCAACCCCTAAGCACAGCTTATTGTTGGGCGTCTACTCCTGTAGTGCCTCAAAGACATTGGCGGAGCGGCAGTAGTCCTCTGAGCGTAGTAATTCTTTATCTCGCTTCTGTTAGGCGCTGCCCCCCCGGCCGTAAAACCCCCCCCATTTTTTTCTGGTTGACCTCGGATCAGGTAGGAATACCCGCTGAACTTAAGCATATTCTGTTAGGCGCTGCCCCCCCGGCCGTAAAACCCCCCCCATTTTTTTCTGGTTGACCTCGGATCAGGTAGGAATACCCGCTGAACTTAAGCATAT	MF327715

a Details of sequence submission on the NCBI database can be found at https://www.ncbi.nlm.nih.gov/nuccore/MF327715.

The fungus was subjected to solid-state fermentation, and the resulting secondary metabolites were extracted and evaluated for antimicrobial and antioxidant activities. The crude fungal extract showed moderate antibacterial activity at 1 mg/mL, producing an IZD of 2 mm against *Bacillus subtilis* and *Salmonella typhi.* However, it was inactive against the tested fungal strains (Table 3). The extract also exhibited average antioxidant activity (48% inhibition) at 500 μg/mL (Table 4).

**Table 3:** Antimicrobial Assay Showing the Mean IZDs (mm) ± Standard Error of Mean (SEM) Produced Against Test Organisms

test organisms	fungal extract (1 mg/mL)	positive control ciprofloxacin(5 μg/mL)	negative control DMSO
Staphylococcus aureus	0 ± 0.00	5 ± 0.33	0 ± 0.00
B. subtilis	2 ± 0.00	8 ± 0.00	0 ± 0.00
S. typhi	2 ± 0.00	7 ± 0.33	0 ± 0.00
Escherichia coli	0 ± 0.00	8 ± 0.33	0 ± 0.00
		miconazole (50 μg/mL)	DMSO
Candida albicans	0 ± 0.00	12 ± 0.89	0 ± 0.00
Aspergillus niger	0 ± 0.00	14 ± 0.33	0 ± 0.00

**Table 4:** DPPH Antioxidant Assay

Sample (500 μg/mL)	% inhibition
fungal extract	48
quercetin	93

### 2.2 Structure Elucidation of **1–6**

Compounds **1** and **2** coeluted and were isolated as an inseparable mixture (1:3) in form of an off-white amorphous solid using preparative high performance liquid chromatography (HPLC). The HPLC chromatogram (Figure S1) revealed a single peak that could not be clearly separated. The molecular formulas of **1** and **2** were determined to be C_11_H_12_Cl_2_N_2_O_5_ and C_10_H_12_O_4_ based on their HR-ESI-MS spectra that revealed protonated molecular ion peaks at *m*/*z* 323.0196 [M + H]^+^ and *m*/*z* 197.0808 [M + H]^+^ indicating six and five degrees of unsaturation, respectively. The ^1^H NMR spectral data of **1** and **2** (see Supporting Information Figure S4, Table S1) revealed two different sets of proton signals that could be easily distinguished based on their different integration indices. One set revealed two aromatic resonances at δ_H_ 7.66/8.20 each integrated for two protons and thus indicating the presence of 1,4-disubstituted aromatic ring. In addition, the ^1^H NMR spectrum of **1** revealed one oxygenated methylene group as two diastereotopic protons at δ_H_ 3.63 (dd, *J* = 10.9, 6.0 Hz) and 3.83 (dd, *J* = 10.9, 7.2 Hz) that were correlated via the HSQC spectrum (Figure S8) to sp^3^ carbon signal (δ_C_ 62.2). The ^1^H–^1^H COSY spectrum of **1** (Figure S5) revealed a spin system extending from the diastereotopic methylene protons to two sp^3^ methines (δ_H_ 4.16 and 5.18). Based on the results obtained, a literature search of **1** revealed its chemical identity as 3-(*p*-nitrophenyl)-3-hydroxy-4-dichloromethoxy-isobutanamide, a dichlorinated fungal metabolite solely reported from the soil-derived fungus *Acrodontium salmoneum* and trivially named as acrodontiolamide^36^ that exhibited antifungal activity in particular against phyto- and entomopathogenic fungi.^37^ To the best of our knowledge, this study is the second report of acrodontiolamide from a natural source.

The ^1^H and ^13^C NMR spectrum of **1** and **2** (Figures S4 and S6, Table S1) revealed a second set of proton and carbon signals categorized into three olefinic signals at δ_H_ 6.62 (td, *J* = 6.2, 1.4 Hz; δ_C_ 135.0), δ_H_ 6.24 (d, *J* = 2.1 Hz; δ_C_ 100.0) and δ_H_ 5.63 (d, *J* = 2.1 Hz; δ_C_ 89.2). In addition, three proton resonances were also identified as a hydroxymethylene moiety at δ_H_ 4.35 (dd, *J* = 6.2, 1.2 Hz; δ_C_ 59.6), an olefinic methyl δ_H_ 1.92 (d, *J* = 1.2 Hz; δ_C_ 12.5) and an oxygenated methyl group δ_H_ 3.90 (s; δ_C_ 57.0). A literature search of **2** unravelled its identity as an α-pyrone derivative hydroxypestalopyrone that was reported from an endophytic fungus *Pestalotiopsis microspora*.^38,39^

**Figure 1:**
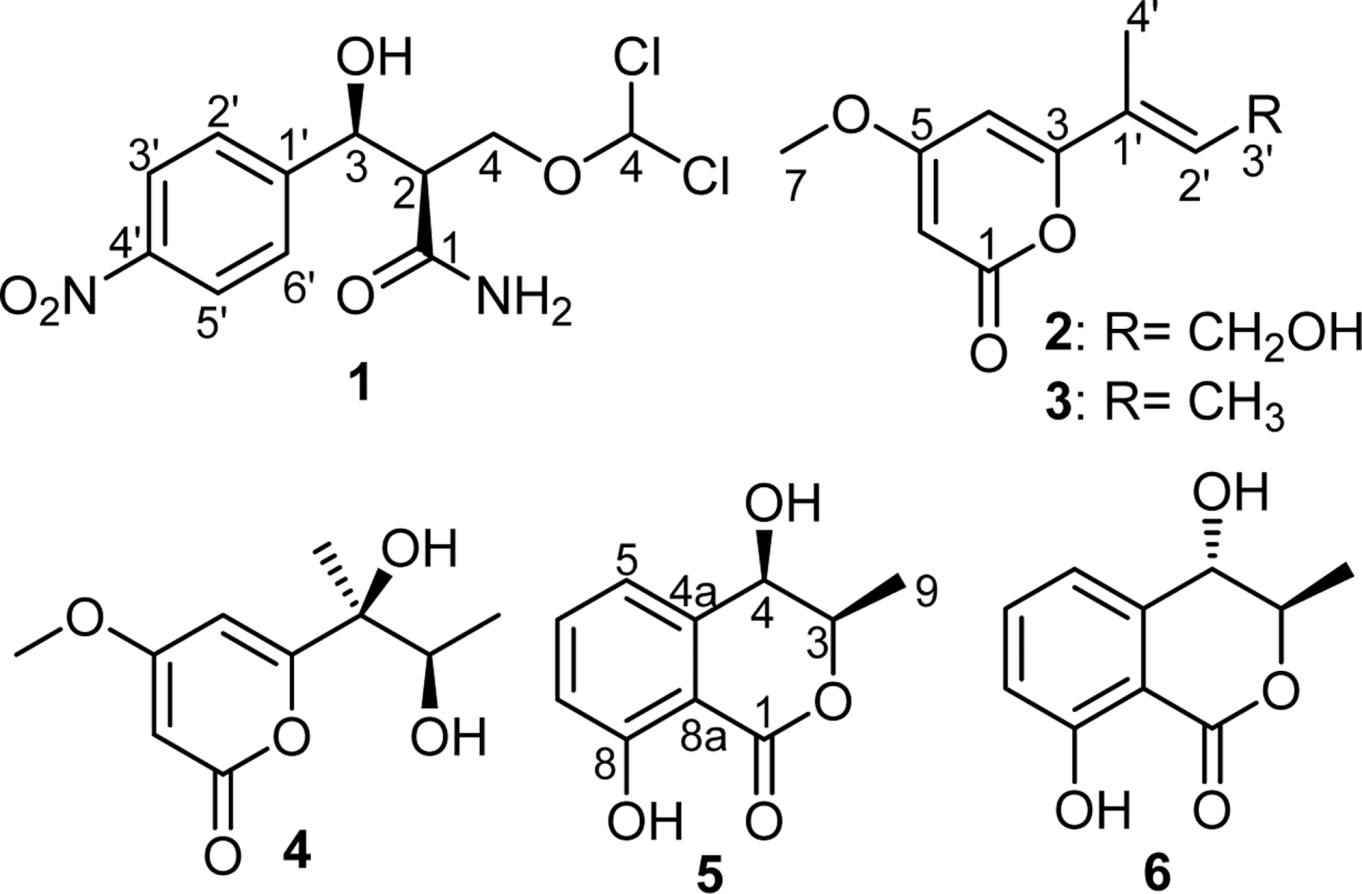
Chemical structures of **1**–**6**.

Further chromatographic separations of the fungal extract afforded four other α-pyrone derivatives (**3**–**6**) (Figure 1) that were identified based on comparing their spectropscopic (1D/2D NMR) and mass spectrometric analyses with the reported literature namely; pestalopyrone (**3**),^38,39^ pestalotiopyrone D (**4**),^40,41^*cis*-4-hydroxymellein (**5**),^42,43^ and *trans*-4-hydroxymellein (**6**).^42,43^

Nigrospora sp. is a fungus in the class Sordariomycetes that can exist as an endophyte, saprophyte, and/or opportunistic pathogen depending upon host and environmental conditions.^44^*Nigrospora oryzae* is known to cause leaf spots on wild rice.^45^

Fungi of the genus Nigrospora have proven to be a rich source of secondary metabolites such as nigrosporolide, nigrosporapyrone, nigrosporins, nigrospoxydons, epoxydon, deoxyabscisic acid, abscisic acid, phaseic acid, pestalopyrone, hydroxypestalopyrone, clavatol, 3-hydroxymethylphenol, and lactones. These compounds exhibit diverse biological properties, including antibacterial, antifungal, anti-inflammatory, antioxidant, cytotoxic, phytotoxic, and plant-growth regulating activities.^38,46,47^

The genus Nigrospora has been isolated from several other plants, like *Moringa oleifera*, *Paraserianthes falcataria*, *Emblica officinalis*, *Combretum dolichopetalum* and *Loranthus micranthus*.^48−51^ Several biologically important compounds have been reportedly isolated from endophytic Nigrospora sp. such as griseofulvin and dechlorogriseofulvin (antifungal), 8-dihydroramulosin (larvicidal), mullein and its derivatives (antimicrobial, antiviral, and phytotoxic), altersolanol derivatives (antimicrobial, hypoglycaemic, anticancer, and cytotoxic), guaijaverin (antiplasmodial and antimicrobial), isoquercetrin, (antioxidant, anti-inflammatory, cardioprotective, anticancer, antiallergic, antiprotozoal and antidiabetic), and hyperin (antimicrobial, antioxidant, antiviral, anti-inflammatory, and anticancer).^49−56^

In this study, a rare chlorinated fungal metabolite, acrodontiolamide (**1**), and five α-pyrone compounds, hydroxypestalopyrone (**2**), pestalopyrone (**3**), pestalotiopyrone D (**4**), *cis*-4-hydroxymellein (**5**), and *trans*-4-hydroxymellein (**6**) were isolated from fermentation extracts of Nigrospora sp., an endophyte of *G. latifolium*. Acrodontiolamide and the α-pyrone compounds reported in this study from Nigrospora sp. have been well reported in the literature to possess a wide range of biological properties, including antimicrobial and/or antioxidant properties. These compounds may have significantly contributed to the antibacterial and antioxidant activities exhibited by the fungal extract (Tables 3 and 4).

The fungal metabolite acrodontiolamide, which has been reported to show inhibitory activity against phytopathogenic and entomopathogenic fungi, was first described in 1993 from a soil-borne fungus, *A. salmoneum*.^36,37^ Similar to many other fungal secondary metabolites, acrodontiolamide has a nitro function attached to an aromatic ring. However, unlike other fungal secondary metabolites, chlorination, which normally affects the aromatic part of a molecule, was located on the compound’s aliphatic moiety. Very few natural product compounds have been reported with monochlorination on the aliphatic C_3_ chain, making this type of halogenation uncommon.^36^ This is the second report of acrodontiolamide from a natural source. The α-pyrone moiety is a key structural feature of a wide range of bioactive metabolites,^57^ exhibiting a variety of biological activities, including antimicrobial, antitumor, and cytotoxic activities.^57−59^ Fairlamb et al. reported the antiproliferative activities of several α-pyrone compounds against cancerous cells, with IC_50_ values ranging from 1.8 to >50 μM.^58^ Calderón-Montan ~o et al. reported the cytotoxicity, due to DNA damage, of 2-pyrone against leukemia cells with an IC_50_ of 1.26 mM.^59^ Another report showed that α-pyrone compounds are effective inhibitors of 20S proteasomes, indicating their anticancer potential, with pestalotiopyrone G showing the strongest activity with an IC_50_ of 1.2 μM.^41^

The isocoumarin derivative, 4-hydroxymellein, represents a class of compounds that has been isolated from several plant species including *Uvaria hamiltonii*.^42^ The mellein derivatives obtained in this study have previously been isolated from other fungi like *Lasiodiplodia theobromae*, *Septoria nordorum*, Xylaria sp., and Phomopsis sp.^60−63^ Mellein-type pentaketides are well-known fungal metabolites with significant biological activity.^62−64^ It revealed algicidal, antibacterial, and antifungal activities against *Chlorella fusca*, *Microbotryum violaceum*, and *Bacillus megaterium*, respectively.^64^

Pestalopyrone and its derivatives, pestaloside and hydroxypestalopyrone, have been isolated from *P. microspora*.^39^ Pestalotiopyrone D, together with other α-pyrone compounds, were previously isolated from *Pestalotiopsis sydowiana*.^41^ These compounds are known phytotoxins that cause chlorosis of plant leaves.^39^ They have also been isolated from *Pestalotiopsis virgatula* and have been reported to have some pharmacological activities.^65,66^ Pestalopyrone, isolated from an endophytic *Phomatospora bellaminuta*, showed activity against *Plasmodium falciparum* with an IC_50_ value of 37 μM. This compound remains a promising prototype molecule for the development of antimalarial drugs.^58^

## 3 Conclusions

This study describes the isolation and structure elucidation of a rare chlorinated fungal metabolite, acrodontiolamide, and five α-pyrone compounds (hydroxypestalopyrone, pestalopyrone, pestalotiopyrone D, *cis*-4-hydroxymellein, and *trans*-4-hydroxymellein), from Nigrospora sp., an endophytic fungus derived from the leaves of *G. latifolium*. These compounds possess a wide range of biological properties, including antimicrobial, antitumor, and cytotoxic activities, making them promising candidates for exploration in drug development.

## 4 Materials and Methods

### 4.1 Plant Collection

Fresh leaves of *G. latifolium* were harvested from a farm at Ifite Dunu town in Dunukofia Local Government Area of Anambra State (coordinates: 6°20′N 7°00′E), southeastern Nigeria. The plant material was authenticated by a plant taxonomist, and a voucher specimen (PCG474/A/006) deposited in the herbarium of the Department of Pharmacognosy and Traditional Medicine at Nnamdi Azikiwe University, Nigeria.

### 4.2 Fungal Isolation and Identification

Endophytic fungal isolation was carried out using a previously described method .^2^ The plant leaves were carefully washed in running tap water before being cut into small fragments each of about 1 cm^2^. The leaf fragments were surface-sterilized by complete immersion in a 2% sodium hypochlorite solution, followed by 70% ethanol, and then rinsed in sterile water. The sterilized leaf fragments were placed onto Petri dishes containing malt extract agar (MEA) supplemented with chloramphenicol. The Petri dishes were incubated at 28 °C for 2–3 days, during which fungal growth from the leaf fragments was observed. To obtain pure colonies, hyphal tips from distinct colonies growing from leaf segments were transferred onto fresh MEA plates. Using a previously described method,^67^ with modifications, the fungus was identified by amplifying and sequencing the internal transcribed spacer (ITS) regions of the fungal rDNA (rDNA) and comparing the resulting sequence data to those in the NCBI GenBank database. Details of the fungal identification are provided below.

### 4.3 Molecular Taxonomic Identification Protocol

The fungal genomic DNA was extracted and purified directly from fresh, axenic mycelia using the Quick-DNA Fungal/Bacterial Miniprep Kit (Zymo Research Corporation, USA) as described in the manufacturer’s instructions. DNA amplification by PCR was performed using ‘Hot StarTaq Master Mix Taq polymerase and the primer pair ITS1 and ITS4 (Invitrogen, Germany) in an iCycler (Bio-Rad, Germany) thermocycler using the following program: (i) initial denaturation 95 °C, 15 min; (ii) denaturation 95 °C, 1 min; (iii) annealing 56 °C, 1 min; (iv) extension 72 °C, 1 min; (v) final extension 72 °C, 10 min. Steps (ii–iv) were repeated 35 times. The fungal DNA was placed in a 0.2 mL PCR tube containing 25 μL HotStarTaq Master Mix, 1.5 μL primer mix (10 pmol/μL each), 1 μL of 10–100 ng template DNA, and water until 50 μL. The negative control, which contained all the above listed except fungal DNA, was also prepared. Sequences for the ITS1 and ITS4 primers are shown in Table 1. After PCR, 50 μL of the PCR product was mixed with 10 μL of 6X gel loading dye and 25 μL of sample was loaded onto an agarose gel [1% agarose (Biozym Scientific GmbH) in 1/10 TBE buffer, 12 μL SYBR Safe DNA gel stain (supplied as a 10,000× concentrate in DMSO) (Invitrogen)]. One well was loaded with 10 μL Quick-Load 100 bp DNA Ladder (New England Biolabs, Inc. USA). Electrophoresis was then run at 75 V for 45 min. After electrophoresis, the gel was viewed on a UV transilluminator to confirm that the PCR had been successfully carried out and that the PCR product was of the correct size, approximately 550 bp, by comparing it with the DNA ladder. The DNA fragment was then excised from the agarose gel with a clean, sharp scalpel. Pure DNA was obtained from the DNA fragment using the Zymoclean Gel DNA Recovery Kit (Zymo Research Corp, USA) according to the manufacturer’s protocol. The DNA sample and ITS1 primer (7.5 μL DNA + 2.5 μL ITS1) were placed in a sterile 2 mL tube and submitted to GATC Biotech AG (Köln, Germany) for direct sequencing using Sanger sequencing technology. After sequencing, BioEdit (version 7.2.5) was used to view the sequencing chromatogram and check for quality. The sequence was then copied in FASTA format and pasted on query box in the NCBI BLAST Web site (https://blast.ncbi.nlm.nih.gov/Blast.cgi).

### 4.4 Fungal Fermentation and Extraction of Secondary Metabolites

The endophytic fungus was subjected to solid-state fermentation in five 1 L Erlenmeyer flasks, each containing autoclaved rice medium (100 g of rice in 100 mL of distilled water), as previously described.^2,3^ Previous studies have validated the use of rice medium for fungal fermentation and bioprospecting of novel metabolites.^1,10,69^ This approach offers several advantages: first, solid-state fermentation in rice medium promotes high yields of microbial secondary metabolites. Second, the rice medium itself does not contribute significantly to the fungal secondary metabolite profile, making it easier to identify and isolate the distinct metabolites produced by the fungus. The flask was inoculated with 3 mm agar blocks containing the fungus and incubated at 28 °C for 30 days. At the end of the fermentation, the fungal secondary metabolites were extracted from each fermentation flask with 500 mL of ethyl acetate and concentrated under reduced pressure at 40 °C using a rotary evaporator.

### 4.5 Detection and Purification of Metabolites

The total fungal crude extract (5.6 g) derived from the five fermentation flasks was fractionated using vacuum liquid chromatography on silica gel 60 (Merck) with stepwise gradient elution, achieved by gradually increasing the polarity of the solvent system *n*-hexane (*n*-hex) and ethyl acetate (EtOAc) combinations in the ratios of 100:0, 70:30, 50:50, 30:70, and 0:100. Fractions were monitored using thin layer chromatography on silica gel 60 F_254_ plates (Merck) with UV detection at 254 and 365 nm. Fractions containing metabolites [*n*-hex/EtOAc (50:50, 30:70, and 0:100)] were further purified using semipreparative HPLC on a Merck-Hitachi HPLC system. The system was equipped with an L-7400 UV detector, L-7100 pump, and 300 × 8 mm Eurospher-100 C_18_ column (Knauer, Germany), with gradient or isocratic methanol (MeOH) and water (H_2_O) mixture as the mobile phase (flow rate: 5 mL/min). Semipreparative HPLC of the fractions yielded the following compounds: *n*-hex/EtOAc 50:50 (1.6 g)—**5** (2.3 mg) and **6** (3.2 mg); *n*-hex/EtOAc 30:70—(1.2 g): **3** (2.1 mg); *n*-hex/EtOAc 0:100 (1.9 g)—**1**/**2** (1.5 mg), and **4** (1.2 mg).

Purity of the isolated compounds was assessed using analytical HPLC on a P580 HPLC system (Dionex Softron GmbH) equipped with a P580A LPG pump, UVD340s photodiode array detector (Dionex Softron GmbH), and a 4 × 125 mm Eurospher-10 C_18_ separation column (Knauer, Germany) with UV detection at 235 nm. 1D and 2D NMR spectra of the compounds were recorded in deuterated chloroform or methanol, using Bruker 300 and Bruker Avance III 600 spectrometers. The spectra and their chemical shifts were referenced relative to residual solvent signals, respectively. Mass spectrometry measurements involved an HP Agilent 1100 HPLC-Thermo Quest Finnigan LCQ Deca XP LC/MS system and HR-ESI-MS UHR-QTOF maXis 4G (Bruker Daltonics). All spectroscopic measurements were performed with spectral-grade solvents.

#### 4.5.1 Acrodontiolamide (**1**)

Off-white amorphous solid; UV (MeOH) λ_max_ at 220.1, 309.8 nm; NMR data (^1^H NMR: 600 MHz, ^13^C NMR: 150 MHz in methanol-*d*_4_, see Supporting Information: Table S1) comparable to the reported literature;^36,37^ HR-ESI-MS *m*/*z* 323.0192 [M + H]^+^ (calcd 323.0196 for C_11_H_13_Cl_2_N_2_O_5_^+^, see Supporting Information: Figure S3).

#### 4.5.2 Hydroxypestalopyrone (**2**)

Off-white amorphous solid; UV (MeOH) λ_max_ at 220.1, 309.8 nm; NMR data (^1^H NMR: 600 MHz, ^13^C NMR: 150 MHz in methanol-*d*_4_, see Supporting Information: Table S1) comparable to the reported literature;^38,39^ HR-ESI-MS *m*/*z* 197.0807 [M + H]^+^ (calcd 197.0808 for C_10_H_13_O_4_^+^, see Supporting Information: Figure S3).

#### 4.5.3 Pestalopyrone (**3**)

Light yellow amorphous solid; UV (MeOH) λ_max_ at 222.1, 256.8, 311.4 nm; ^1^H NMR (300 MHz in methanol-*d*_4_, see Supporting Information: Table S2) comparable to the reported literature;^38,39^ HR-ESI-MS *m*/*z* 181.0859 [M + H]^+^ (calcd 181.0859 for C_10_H_13_O_3_^+^, see Supporting Information: Figure S13).

#### 4.5.4 Pestalotiopyrone D (**4**)

Colorless oil; UV (MeOH) λ_max_ at 205.1, 281.1 nm. ^1^H NMR (300 MHz in methanol-*d*_4_, see Supporting Information: Table S3) comparable to the reported literature;^40,41^ HR-ESI-MS *m*/*z* 215.0916 [M + H]^+^ (calcd 215.0914 for C_10_H_15_O_5_^+^, see Supporting Information: Figure S18).

#### 4.5.5 *cis*-4-Hydroxymellein (**5**)

White amorphous solid; UV (MeOH) λ_max_ 217.2, 244.1, 313.0 nm; ^1^H NMR (300 MHz in chloroform-*d*, see Supporting Information: Table S4) comparable to the reported literature;^42,43^ LR-ESI-MS *m*/*z* 193.3 [M-H]^−^ and 195 [M + H]^+^ (see Supporting Information: Figure S23).

#### 4.5.6 *trans*-4-Hydroxymellein (**6**)

White needle-like crystals; UV (MeOH) λ_max_ 216, 246, 314 nm; ^1^H NMR (300 MHz in chloroform-*d*, see Supporting Information: Table S5) comparable to the reported literature;^42,43^ LR-ESI-MS *m*/*z* 195.2 [M + H]^+^ (see Supporting Information: Figure S29).

### 4.6 Bioassays

#### 4.6.1 Antimicrobial Assay

The agar-well diffusion method described by Akpotu et al.^4^ and Enyi et al.^5^ was used to assess the antimicrobial property of the crude fungal extract. The extract was tested against *S. aureus*, *B. subtilis*, *S. typhi*, and *E. coli*, *A. niger* and *C. albicans*. These laboratory-derived strains were obtained from the Department of Pharmaceutical Microbiology and Biotechnology, Nnamdi Azikiwe University, Nigeria. A 1 mg/mL solution of the fungal extract was prepared in 100% (v/v) dimethyl sulfoxide (DMSO). Then, 20 mL of molten Mueller–Hinton agar (for bacteria) and Sabouraud dextrose agar (for fungi) were poured into sterile Petri dishes (90 mm) and allowed to solidify. Standardized concentrations (McFarland 0.5) of overnight cultures were swabbed aseptically onto the agar plates. Subsequently, 6 mm wells were made in the agar using a sterile metal cork-borer. Under aseptic conditions, 20 μL of the extract and controls were added to each well. Positive controls for the antibacterial and antifungal tests were ciprofloxacin (5 μg/mL) and miconazole (50 μg/mL), respectively. DMSO (100% v/v) served as the negative control in both tests. The plates were kept at room temperature for 1 h to allow the agents to diffuse into the agar medium before incubating at 37 °C for 24 h (Mueller–Hinton agar) and 25 °C for 48 h (Sabouraud dextrose agar), respectively. Inhibition zone diameters (IZDs) were calculated by subtracting the size of the well (6 mm) from the measured zones of inhibition. This procedure was conducted in triplicate, and the mean IZDs ± standard error of mean (SEM) were calculated and recorded.

#### 4.6.2 Antioxidant Assay

The antioxidant property of the crude fungal extract was also evaluated using the 1,1-diphenyl-2-picrylhydrazyl (DPPH) free radical scavenging test method previously described by Abba et al.^6^ Stock solutions of the fungal extract (1000 μg/mL), positive control (quercetin, 1000 μg/mL) and DPPH solution (0.2 mM) were prepared in MeOH. The sample and DPPH solution were mixed in equal volumes, resulting in final concentrations of 500 μg/mL and 0.1 mM, respectively. The mixture was incubated for 30 min in the dark at room temperature. A UV–vis spectrophotometer was used to measure absorbance at 517 nm using MeOH as a blank. The free radical scavenging effect of the samples was computed using the formula: 
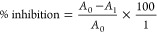
where *A*_0_: absorbance of blank, *A*_1_: absorbance of sample.

### 4.7 Statistical Analysis

The mean IZDs ± standard error of mean (SEM) values obtained from the antimicrobial assay were calculated using Microsoft Excel version 16.82.

## Data Availability

All relevant data are included within the article and its Supporting Information. Sequencing raw data are publicly available via the NCBI GenBank database under the accession number MF327715 (https://www.ncbi.nlm.nih.gov/nuccore/MF327715).

## Supporting Information Available

The HPLC, NMR and mass spectral data of the compounds (PDF)
