# Supplementary material for: Rare Chlorinated Fungal Metabolite and Alpha-Pyrones from an Endophytic Fungus Nigrospora sp
Source: ACS Omega. 2025 Feb 3;10(6):5722–9. doi: 10.1021/acsomega.4c09190 (PMC11840770; doi:10.1021/acsomega.4c09190)
Supplement: Supplementary file 1 — ao4c09190_si_001.pdf [file ao4c09190_si_001.pdf]

SUPPLEMENTARY MATERIAL

A Rare Chlorinated Fungal Metabolite and Alpha-Pyrones from an Endophytic Fungus *Nigrospora* sp.

Chika C. Abba,[†] Peter M. Eze,[‡,§]* Sherif S. Ebada,[Δ]* Nchekwube K. Eze,[¶] Peter Proksch,[∇] Nicole Teusch,[∇] Festus B. C. Okoye,[†] and Chukwueweniwe J. Eboka[†,⊥]

[†] Department of Pharmaceutical and Medicinal Chemistry, Nnamdi Azikiwe University, Awka, Nigeria.

[‡] School of Biological Sciences, Queen's University Belfast, Northern Ireland, United Kingdom.

[§] Department of Environmental Health Science, Nnamdi Azikiwe University, Awka, Nigeria.

[Δ] Department of Pharmacognosy, Faculty of Pharmacy, Ain Shams University, Cairo, Egypt.

[¶] Department of Pharmaceutical Microbiology and Biotechnology, Nnamdi Azikiwe University, Awka, Nigeria.

[∇] Institute of Pharmaceutical Biology and Biotechnology, Heinrich Heine University, Düsseldorf, Germany.

[⊥] Department of Pharmaceutical Chemistry, University of Benin, Benin City, Nigeria.

* Corresponding authors:
Email: p.eze@qub.ac.uk (P. M. Eze), sherif_elsayed@pharma.asu.edu.eg (S. S. Ebada)

Abstract

Endophytic microorganisms have been recognized as potential sources of new chemical entities with applications in the pharmaceutical, biotechnology, agricultural, and other industries. This study investigated the secondary metabolites produced by an endophytic *Nigrospora* sp. isolated from the Nigerian plant, *Gongronema latifolium.* Standard procedures were followed for fungal isolation, taxonomic identification, fermentation, and extraction of secondary metabolites. The antioxidant and antimicrobial properties of the fungal extract were assessed using the 1,1-diphenyl-2-picrylhydrazyl (DPPH) antioxidant assay and the agar-well diffusion assay, respectively. Various chromatographic and spectroscopic techniques were used to isolate, purify, and characterize compounds from the fungal extract. At 500 µg/mL, the fungal crude extract showed average antioxidant activity with a 48% inhibition. The extract also demonstrated moderate antimicrobial activity at 1 mg/mL against *Bacillus subtilis* and *Salmonella typhi*, with an inhibition zone diameter of 2 mm produced against both test strains. The fungal extract yielded six compounds, including the rare, chlorinated metabolite, acrodontiolamide (**1**), and five α-pyrone derivatives: hydroxypestalopyrone (**2**), pestalopyrone (**3**), pestalotiopyrone D (**4**), *cis*-4-hydroxymellein (**5**), and its *trans*-isomer (**6**). Interestingly, this is the second report of acrodontiolamide (**1**) in nature, after its first report in 1993. These compounds possess a wide range of known biological activities, including antimicrobial, antitumor, and cytotoxic effects, valorizing their potential in drug development.

Keywords: Acrodontiolamide, pyrones, *Nigrospora* sp., endophytic fungi, secondary metabolites

Table of Contents

#	Content	Page
**1**	Figure S1. HPLC chromatogram of compounds **1**/**2**.	**S4**
**2**	Figure S2. UV spectrum of compounds **1**/**2**.	**S4**
**3**	Figure S3. HR-ESI-MS of compounds **1**/**2**.	**S5**
**4**	Figure S4. ^{1}H NMR spectrum of **1**/**2** at 600 MHz in methanol-d_4.	**S5**
**5**	Figure S5. ^{1}H–^{1}H COSY spectrum of **1**/**2** at 600 MHz in methanol-d_4.	**S6**
**6**	Table S1. ^{1}H and ^{13}C NMR Data of **1**/**2**.	**S6**
**7**	Figure S6. ^{13}C NMR spectrum of **1**/**2** at 150 MHz in methanol-d_4.	**S7**
**8**	Figure S7. DEPT-135 spectrum of **1**/**2** at 150 MHz in methanol-d_4.	**S7**
**9**	Figure S8. HSQC spectrum of **1**/**2** at 600 MHz in methanol-d_4.	**S8**
**10**	Figure S9. HMBC spectrum of **1**/**2** at 600 MHz in methanol-d_4.	**S8**
**11**	Figure S10. ROESY spectrum of **1**/**2** at 600 MHz in methanol-d_4.	**S9**
**12**	Figure S11. HPLC chromatogram of compound **3**.	**S9**
**13**	Figure S12. UV spectrum of compound **3**.	**S9**
**14**	Figure S13. HR-ESI-MS of **3**.	**S10**
**15**	Figure S14. ^{1}H NMR spectrum of **3** at 300 MHz in methanol-d_4.	**S10**
**16**	Figure S15. ^{1}H–^{1}H COSY spectrum of **3** at 300 MHz in methanol-d_4.	**S11**
**17**	Table S2. ^{1}H NMR Data of **3**.	**S11**
**18**	Figure S16. HPLC chromatogram of compound **4**.	**S11**
**19**	Figure S17. UV spectrum of compound **4**.	**S12**
**20**	Figure S18. HR-ESI-MS of **4**.	**S12**
**21**	Figure S19. ^{1}H NMR spectrum of **4** at 300 MHz in methanol-d_4.	**S13**
**22**	Figure S20. ^{1}H–^{1}H COSY spectrum of **4** at 300 MHz in methanol-d_4.	**S13**
**23**	Table S3. ^{1}H NMR Data of **4**.	**S14**
**24**	Figure S21. HPLC chromatogram of compound **5**.	**S14**
**25**	Figure S22. UV spectrum of compound **5**.	**S14**
**26**	Figure S23. LC-MS of compound **5**.	**S15**
**27**	Figure S24. ^{1}H NMR spectrum of **5** at 300 MHz in methanol-d_4.	**S15**
**28**	Figure S25. ^{1}H NMR spectrum of **5** at 300 MHz in chloroform-*d*.	**S16**
**29**	Figure S26. ^{1}H–^{1}H COSY spectrum of **5** at 300 MHz in methanol-d_4.	**S16**
**30**	Table S4. ^{1}H NMR data of **5**.	**S17**
**31**	Figure S27. HPLC chromatogram of compound **6**.	**S17**
**32**	Figure S28. UV spectrum of compound **6**.	**S17**
**33**	Figure S29. HR-ESI-MS of compound **6**.	**S18**
**34**	Figure S30. ^{1}H NMR spectrum of **6** at 300 MHz in methanol-d_4.	**S18**
**35**	Figure S31. ^{1}H NMR spectrum of **6** at 300 MHz in chloroform-*d*.	**S19**
**36**	Figure S32. ^{1}H–^{1}H COSY spectrum of **6** at 300 MHz in methanol-d_4.	**S19**
**37**	Figure S33. ^{1}H–^{1}H COSY spectrum of **6** at 300 MHz in chloroform-*d*.	**S20**
**38**	Table S5. ^{1}H NMR Data of **6**.	**S20**

DATA FOR COMPOUNDS 1 and 2.

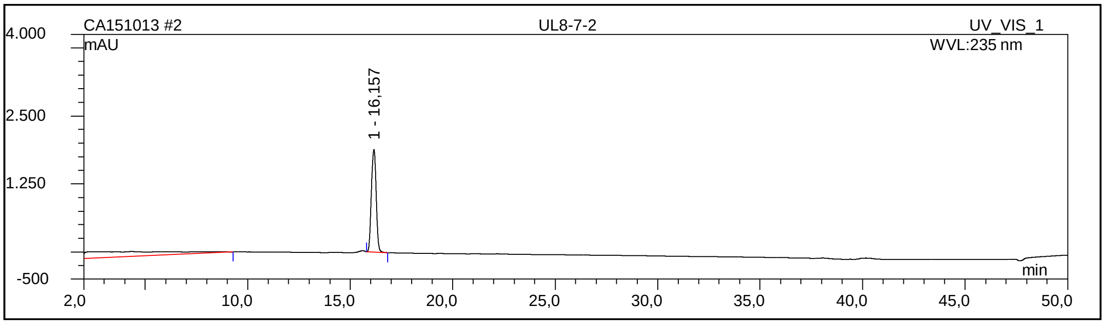

Figure S1. HPLC chromatogram of compounds 1/2.

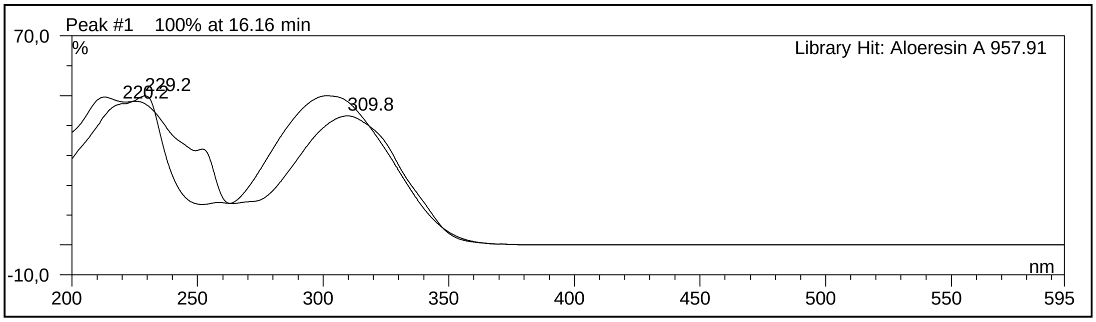

Figure S2. UV spectrum of compounds 1/2.

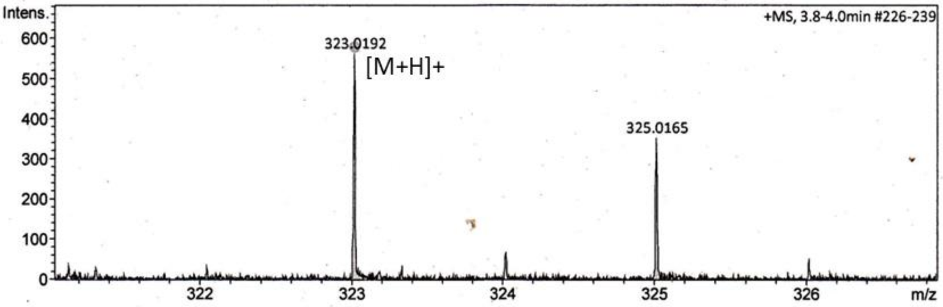

Mass Spectrum SmartFormula Report

Analysis Info

Analysis Name D:\Data\Spektren 2015\Proksch15HR000413.d
Method tune_low.m
Sample Name Chika UL8-7-2 (CH3OH)
Comment

Acquisition Date 11/9/2015 10:28:39 AM
Operator Peter Tommes
Instrument maXis 288882.20213

Acquisition Parameter

Source Type	ESI	Ion Polarity	Positive	Set Nebulizer	0.3 Bar
Focus	Not active	Set Capillary	4000 V	Set Dry Heater	180 °C
Scan Begin	50 m/z	Set End Plate Offset	-500 V	Set Dry Gas	4.0 l/min
Scan End	1500 m/z	Set Collision Cell RF	600.0 Vpp	Set Divert Valve	Source

	Meas. m/z	#	Ion Formula	m/z	err [ppm]	mSigma	# mSigma	Score	rdb	e⁻ Conf	N-Rule
	197.0807	1	C10H13O4	197.0808	0.5	23.5	1	100.00	4.5	even	ok
1 →	323.0192	1	C11H13Cl2N2O5	323.0196	1.1	79.6	1	100.00	5.5	even	ok
		2	C13H4ClN8O	323.0191	-0.4	171.8	2	0.39	15.5	even	ok
		3	C2H3N12O8	323.0191	-0.3	354.5	3	0.00	7.5	even	ok
		4	C17H7O7	323.0186	-1.9	361.7	4	0.00	14.5	even	ok

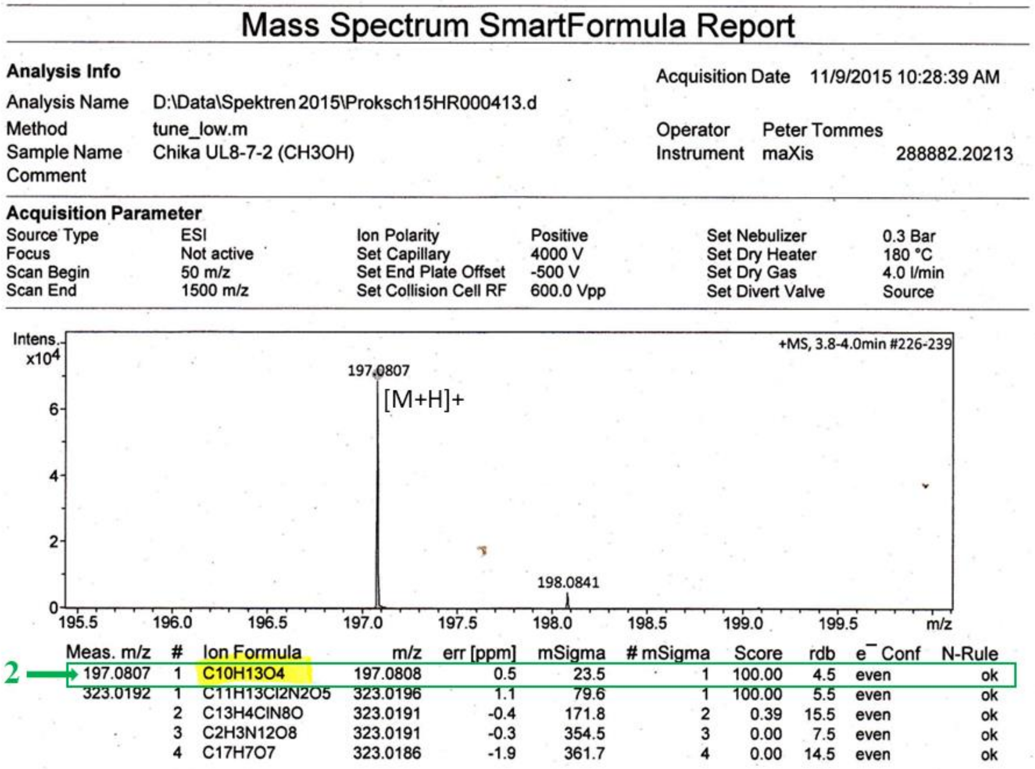

Mass Spectrum SmartFormula Report

Analysis Info

Analysis Name	D:\Data\Spektren 2015\Proksch15HR000413.d	Acquisition Date	11/9/2015 10:28:39 AM
Method	tune_low.m	Operator	Peter Tommes
Sample Name	Chika UL8-7-2 (CH3OH)	Instrument	maXis 288882.20213
Comment			

Acquisition Parameter

Source Type	ESI	Ion Polarity	Positive	Set Nebulizer	0.3 Bar
Focus	Not active	Set Capillary	4000 V	Set Dry Heater	180 °C
Scan Begin	50 m/z	Set End Plate Offset	-500 V	Set Dry Gas	4.0 l/min
Scan End	1500 m/z	Set Collision Cell RF	600.0 Vpp	Set Divert Valve	Source

Meas. m/z	#	Ion Formula	m/z	err [ppm]	mSigma	# mSigma	Score	rdb	e⁻ Conf	N-Rule
2 → 197.0807	1	C10H13O4	197.0808	0.5	23.5	1	100.00	4.5	even	ok
323.0192	1	C11H13Cl2N2O5	323.0196	1.1	79.6	1	100.00	5.5	even	ok
	2	C13H4ClN8O	323.0191	-0.4	171.8	2	0.39	15.5	even	ok
	3	C2H3N12O8	323.0191	-0.3	354.5	3	0.00	7.5	even	ok
	4	C17H7O7	323.0186	-1.9	361.7	4	0.00	14.5	even	ok

Figure S3. HR-ESI-MS of compounds **1**/**2**.

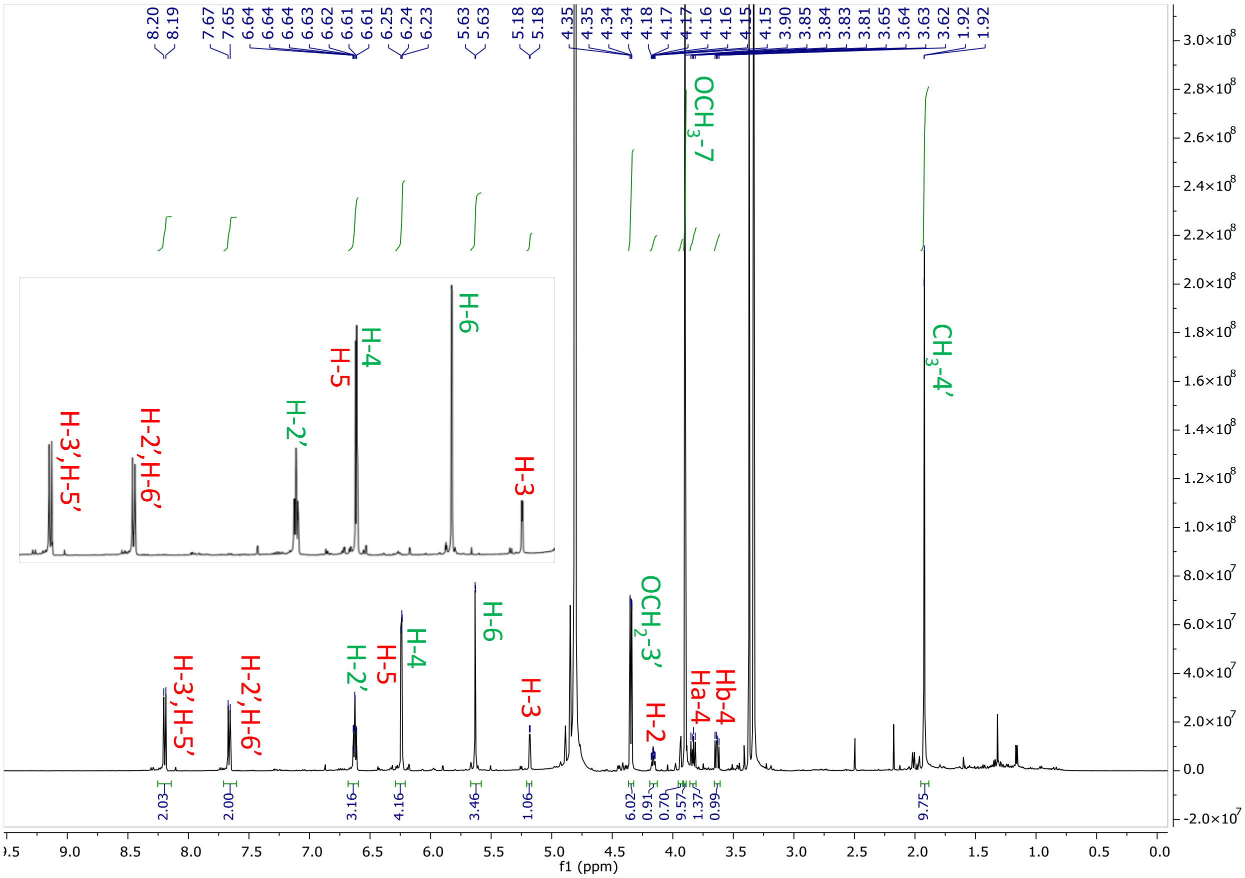

Figure S4. ^{1}H NMR spectrum of **1**/**2** at 600 MHz in methanol-*d*4.

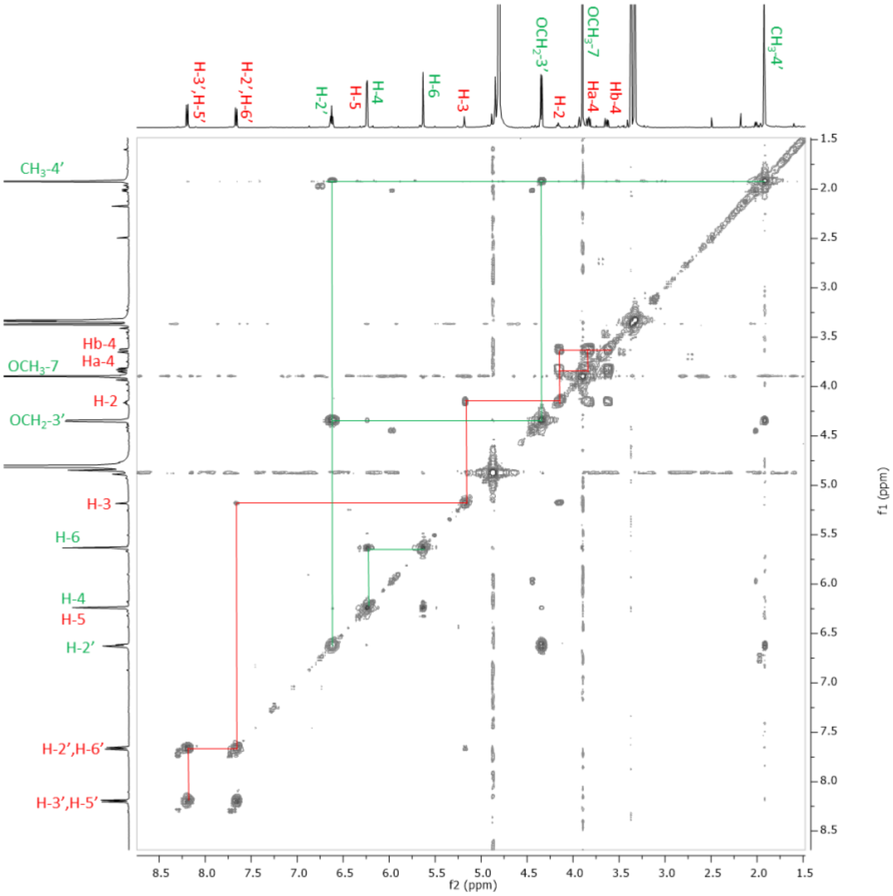

Figure S5. ^{1}H–^{1}H COSY spectrum of **1**/**2** at 600 MHz in methanol-d_4.

Table S1. ^{1}H and ^{13}C NMR Data of **1** and **2**.

	**1**			**2**	
**Position**	**δ_C,[a,c] type**	**δ_H[b] (multi, *J*[Hz])**	**Position**	**δ_C,[a,c] type**	**δ_H[b] (multi, *J*[Hz])**
1	166.6, CO		1	166.6, CO	
2	58.5, CH	4.16 (ddd, 8.8, 6.2, 2.8)	3	161.9, C	
3	71.3, CH	5.18 (d, 2.8)	4	100.0, CH	6.24 (d, 2.1)
4	62.2, CH_2	α 3.63 (dd, 10.9, 6.0)<br>β 3.83 (dd, 10.9, 7.2)	5	173.8, C	
5	67.4, CH	6.25 (s)	6	89.2, CH	5.63 (d, 2.1)
1’	151.7, C		7	57.0, CH_3	3.90 (s, 3H)
2’,6’	128.4, CH	7.66 (d, 8.7, 2H)	1’	128.1, C	
3’, 5’	124.2, CH	8.20 (d, 8.7, 2H)	2’	135.0, CH	6.62 (td, 6.2, 1.4)
4’	148.6		3’	59.6, CH_2	4.35 (dd, 6.2, 1.2)
			4’	12.5, CH_3	1.92 (d, 1.2, 3H)

Measured methanol-d_4 at [a] 150 MHz for ^{13}C and [b] 600 MHz for ^{1}H. [c] Assigned based on HMBC and HSQC spectra.

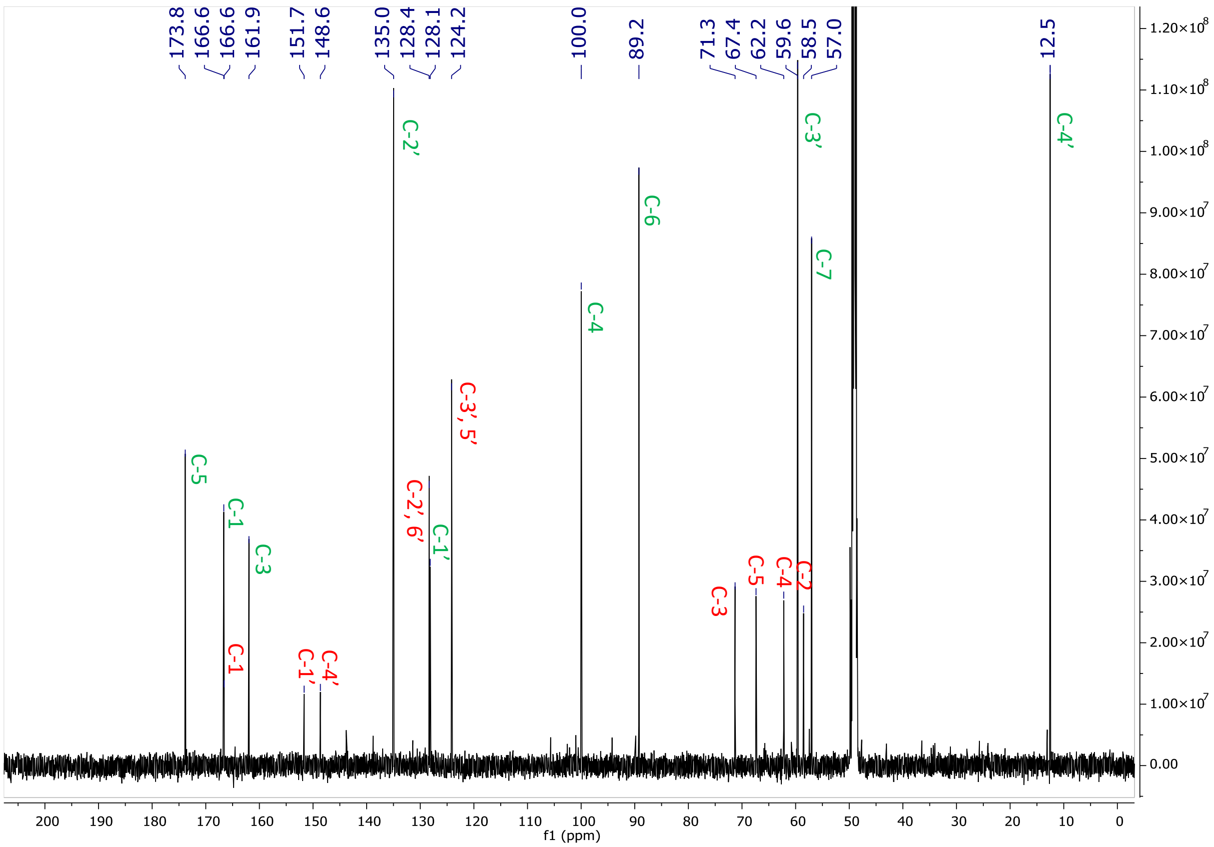

Figure S6. ^{13}C NMR spectrum of **1**/**2** at 150 MHz in methanol-*d*4.

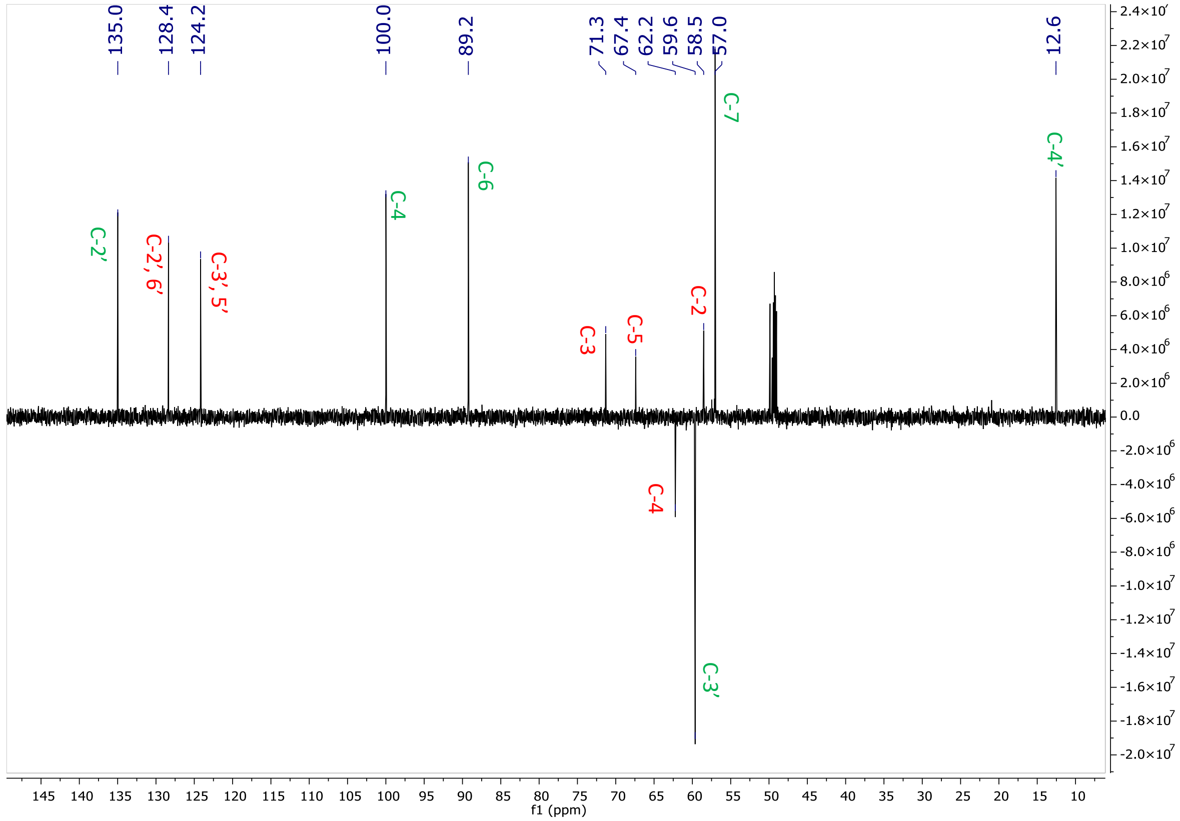

Figure S7. DEPT-135 spectrum of **1**/**2** at 150 MHz in methanol-*d*4.

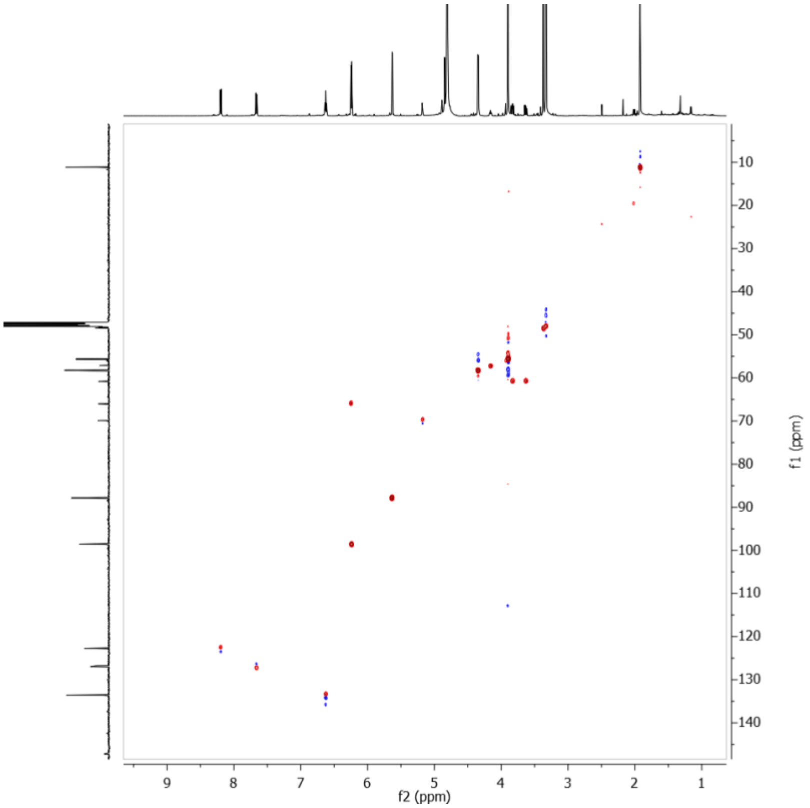

Figure S8. HSQC spectrum of **1**/**2** at 600 MHz in methanol-d_4.

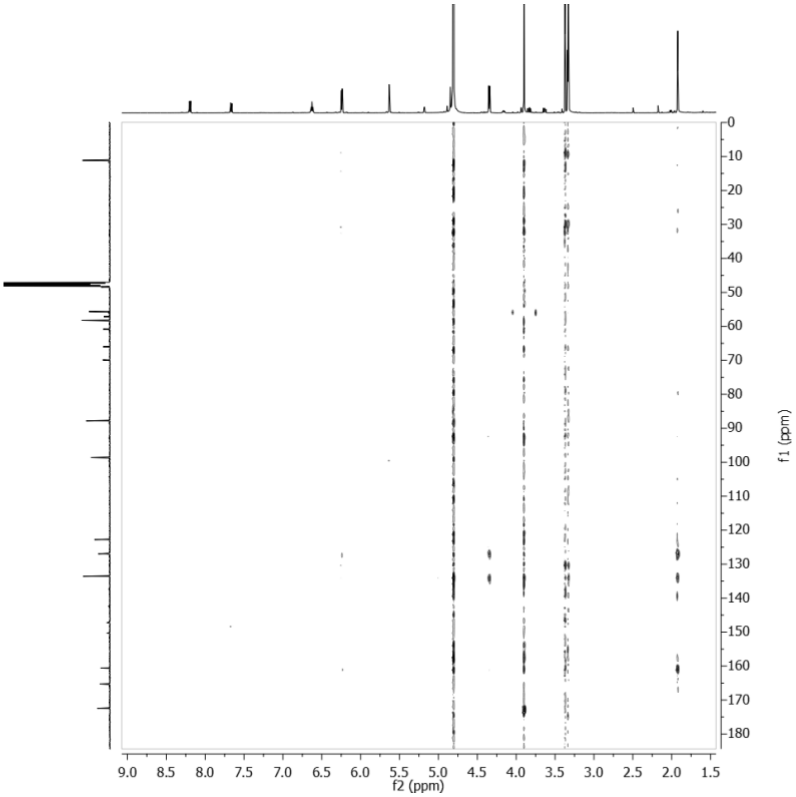

Figure S9. HMBC spectrum of **1**/**2** at 600 MHz in methanol-d_4.

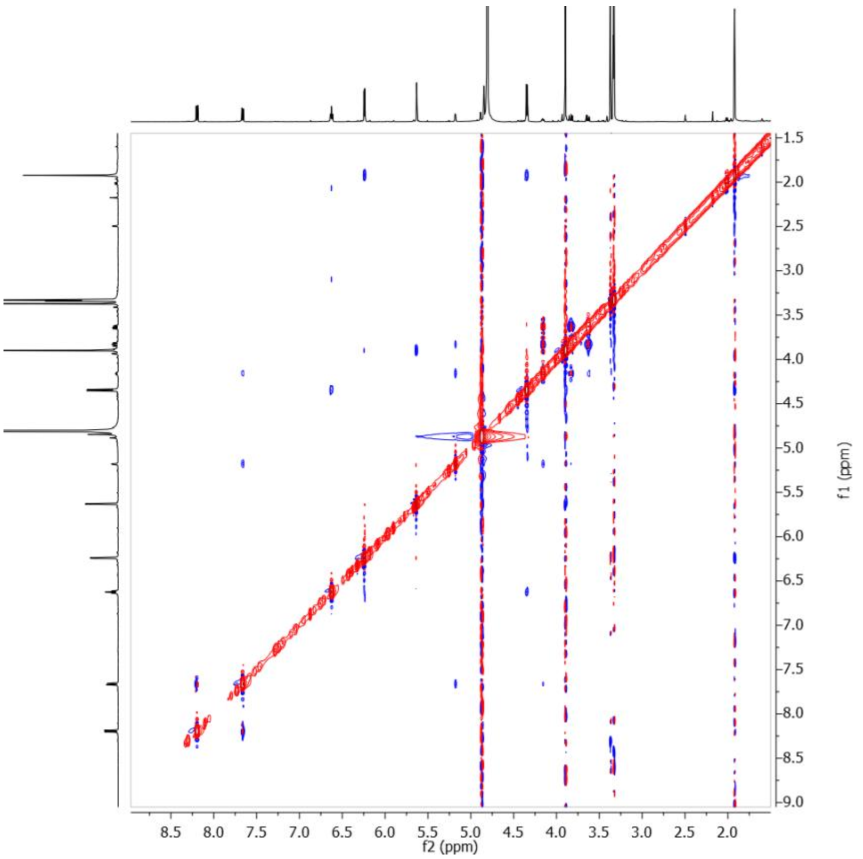

Figure S10. ROESY spectrum of **1**/**2** at 600 MHz in methanol-d_4.

DATA FOR COMPOUND 3

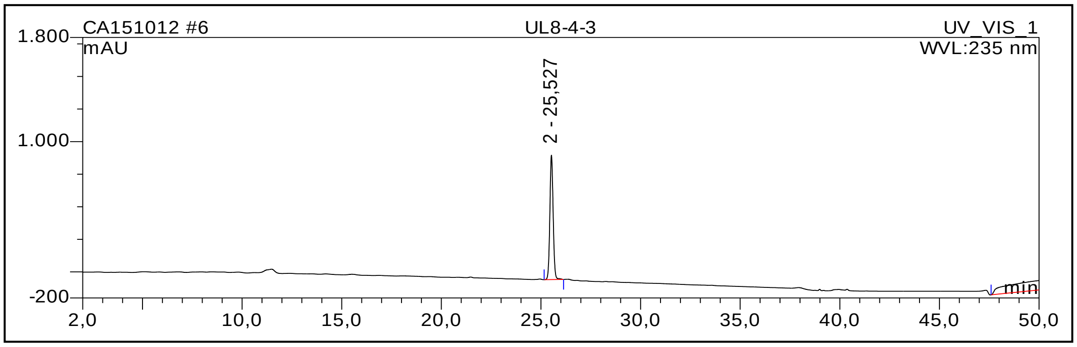

Figure S11. HPLC chromatogram of compound **3**.

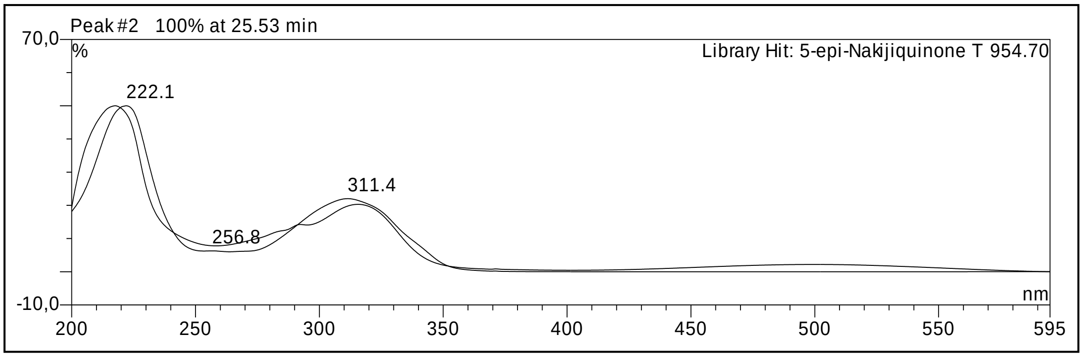

Figure S12. UV spectrum of compound **3**.

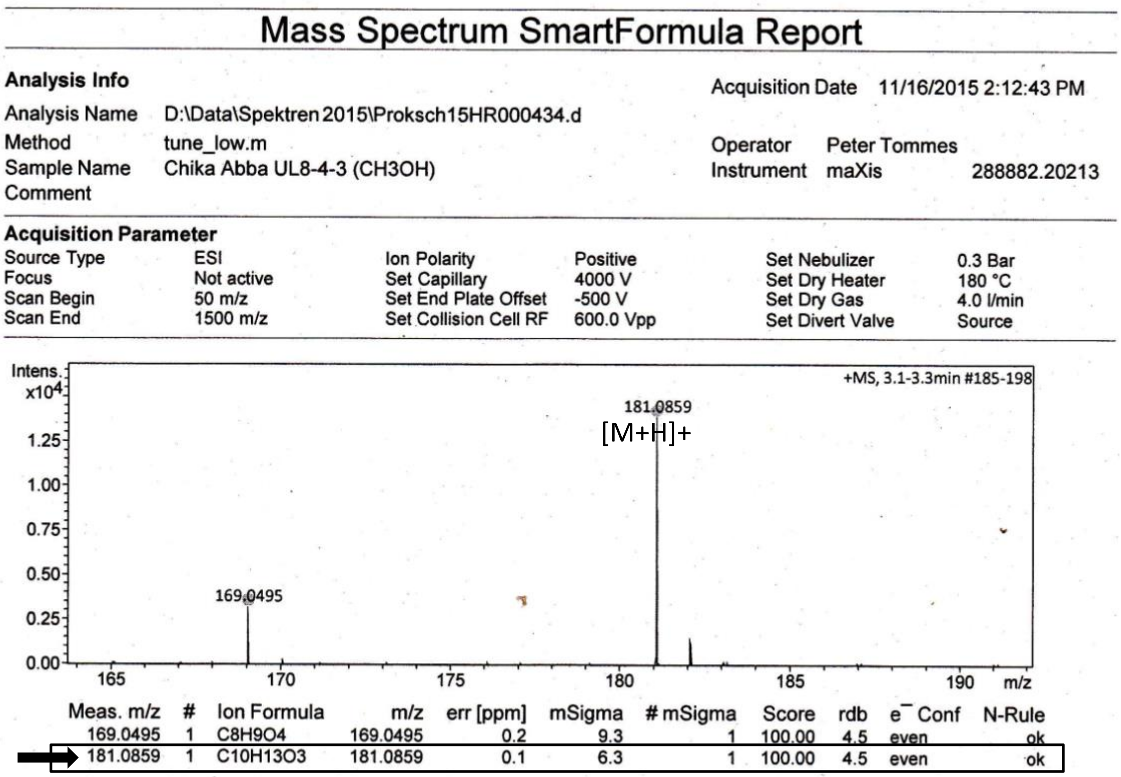

Mass Spectrum SmartFormula Report

Analysis Info

Acquisition Date 11/16/2015 2:12:43 PM

Analysis Name D:\Data\Spektren 2015\Proksch15HR000434.d
Method tune_low.m
Sample Name Chika Abba UL8-4-3 (CH3OH)
Comment

Operator Peter Tommes
Instrument maXis 288882.20213

Acquisition Parameter

Source Type	ESI	Ion Polarity	Positive	Set Nebulizer	0.3 Bar		
Focus	Not active	Set Capillary	4000 V	Set Dry Heater	180 °C		
Scan Begin	50 m/z	Set End Plate Offset	-500 V	Set Dry Gas	4.0 l/min		
Scan End	1500 m/z	Set Collision Cell RF	600.0 Vpp	Set Divert Valve	Source		

Meas. m/z	#	Ion Formula	m/z	err [ppm]	mSigma	# mSigma	Score	rdb	e⁻ Conf	N-Rule
169.0495	1	C8H9O4	169.0495	0.2	9.3	1	100.00	4.5	even	ok
181.0859	1	C10H13O3	181.0859	0.1	6.3	1	100.00	4.5	even	ok

Figure S13. HR-ESI-MS of **3**.

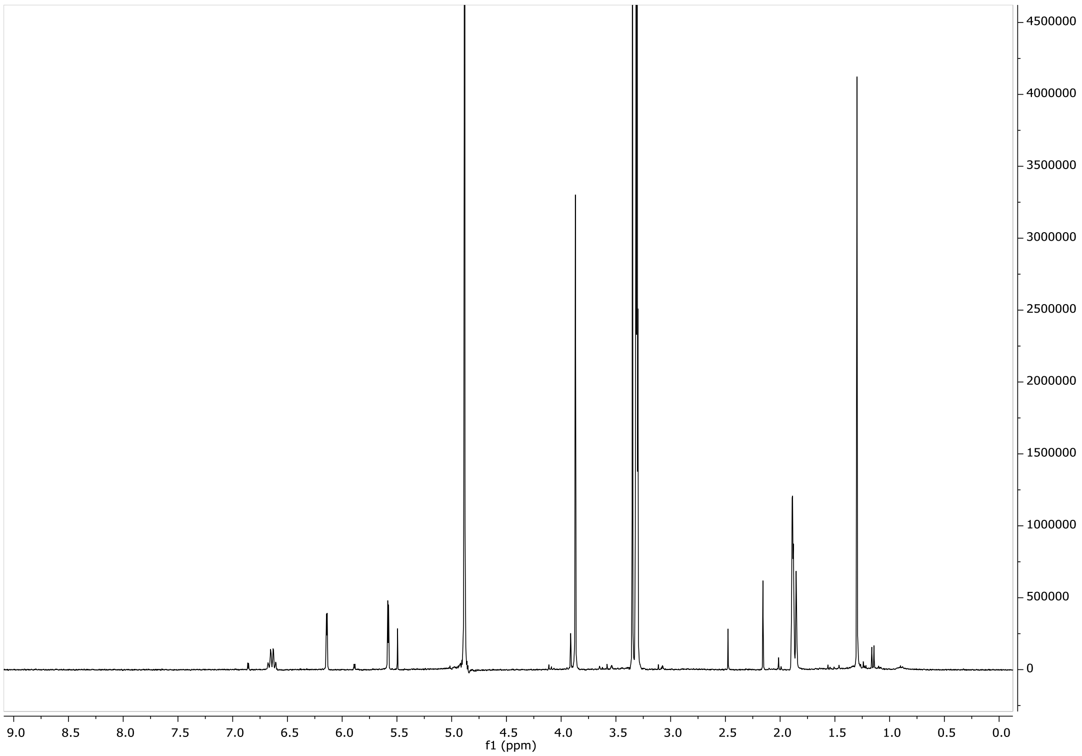

Figure S14. ^{1}H NMR spectrum of **3** at 300 MHz in methanol-d_4.

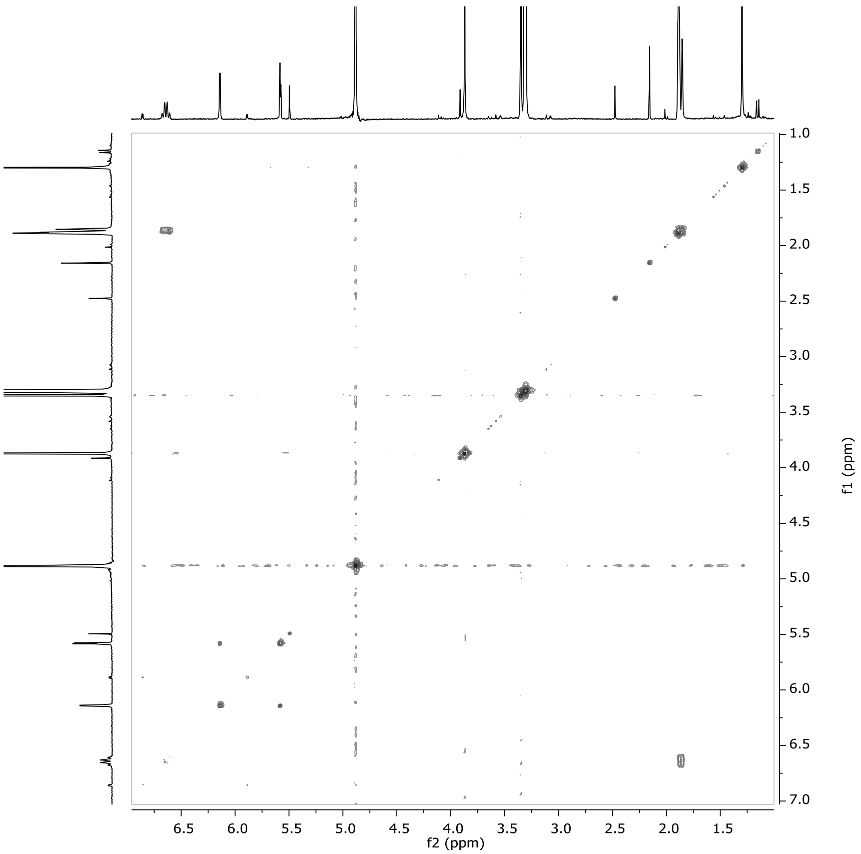

Figure S15. ^{1}H–^{1}H COSY spectrum of **3** at 300 MHz in methanol-d_4.

Table S2. ^{1}H NMR Data of **3**.

**Position**	**δ_H (multi, *J*[Hz])**
4	6.14 (d, 2.1)
6	5.58 (d, 2.1)
7	3.87 (s, 3H)
2’	6.62 (qd, 6.9, 1.0)
3’	1.87 (d, 6.9, 3H)
4’	1.89 (d, 1.0, 3H)

Measured at 300 MHz in methanol-d_4.

DATA FOR COMPOUND 4

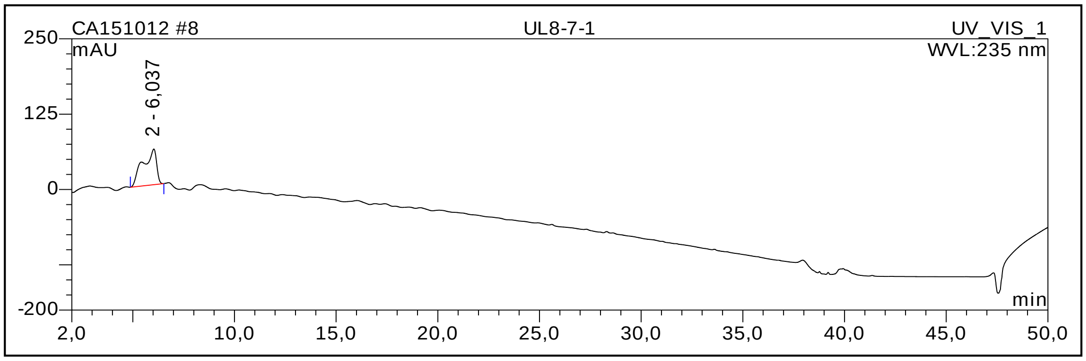

Figure S16. HPLC chromatogram of compound **4**.

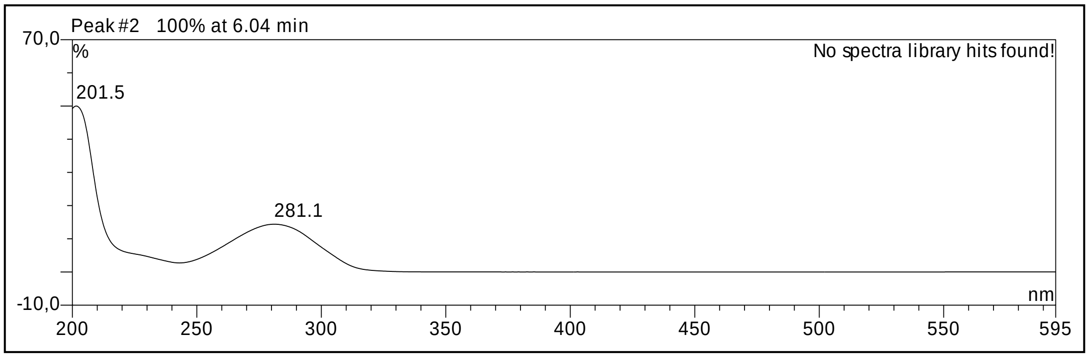

Figure S17. UV spectrum of compound **4**.

Mass Spectrum SmartFormula Report

Analysis Info

Analysis Name	D:\Data\Spektren 2015\Proksch15HR000412.d	Acquisition Date	11/9/2015 10:10:44 AM
Method	tune_low.m	Operator	Peter Tommes
Sample Name	Chika UL8-7-1 (CH3OH)	Instrument	maXis 288882.20213
Comment			

Acquisition Parameter

Source Type	ESI	Ion Polarity	Positive	Set Nebulizer	0.3 Bar
Focus	Not active	Set Capillary	4000 V	Set Dry Heater	180 °C
Scan Begin	50 m/z	Set End Plate Offset	-500 V	Set Dry Gas	4.0 l/min
Scan End	1500 m/z	Set Collision Cell RF	600.0 Vpp	Set Divert Valve	Source

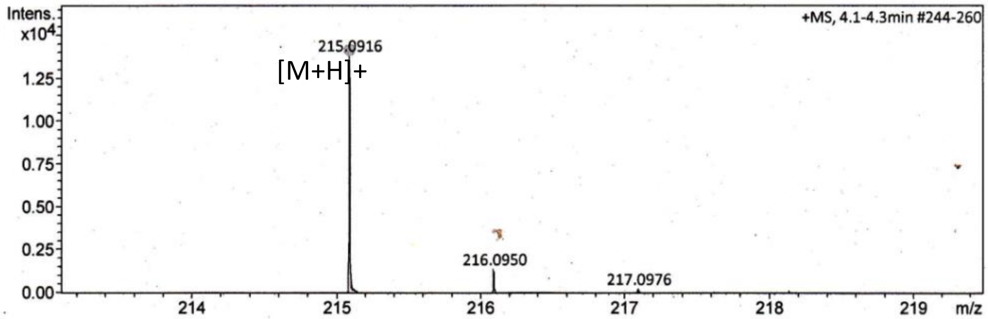

Meas. m/z	#	Ion Formula	m/z	err [ppm]	mSigma	# mSigma	Score	rdb	e⁻ Conf	N-Rule
215.0916	1	C10H15O5	215.0914	-0.7	10.2	1	100.00	3.5	even	ok

Figure S18. HR-ESI-MS of **4**.

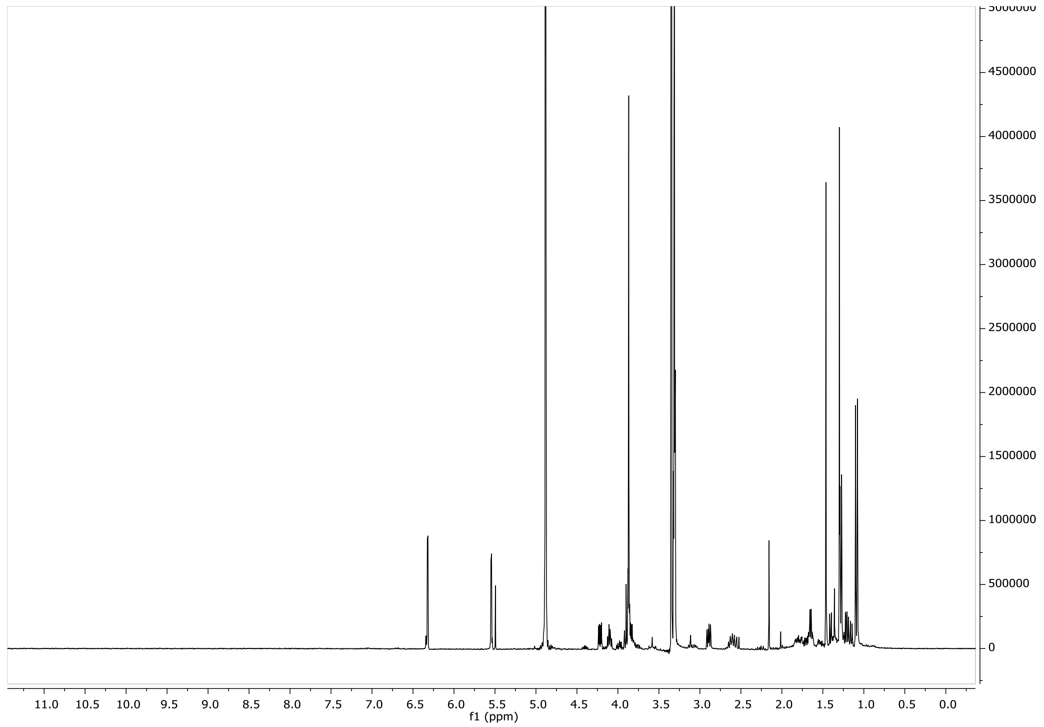

Figure S19. ^{1}H NMR spectrum of **4** at 300 MHz in methanol-d_4.

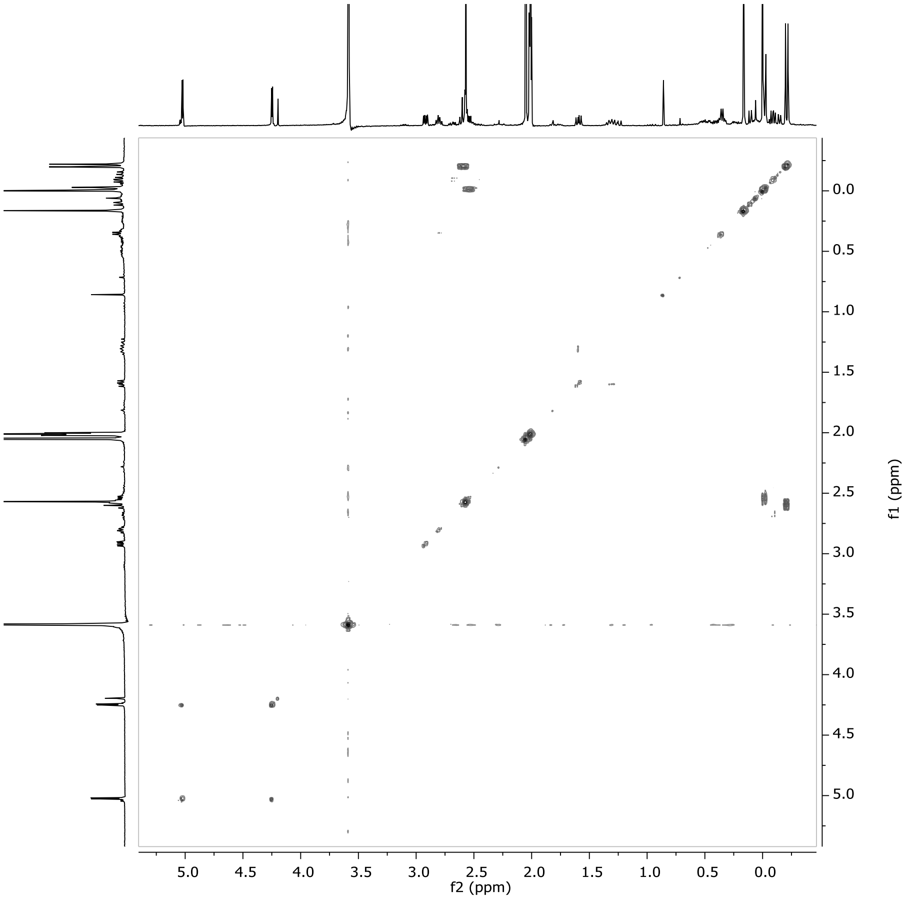

Figure S20. ^{1}H–^{1}H COSY spectrum of **4** at 300 MHz in methanol-d_4.

Table S3. ^{1}H NMR Data of **4**.

	**4**
**Position**	**δ_H (multi, *J*[Hz])**
4	6.32 (d, 2.3)
6	5.55 (d, 2.3)
7	3.87 (s, 3H)
2'	3.89 (q, 6.3)
3'	1.09 (d, 6.3, 3H)
4'	1.46 (s, 3H)

Measured at 300 MHz in methanol-d_4.

DATA FOR COMPOUND 5

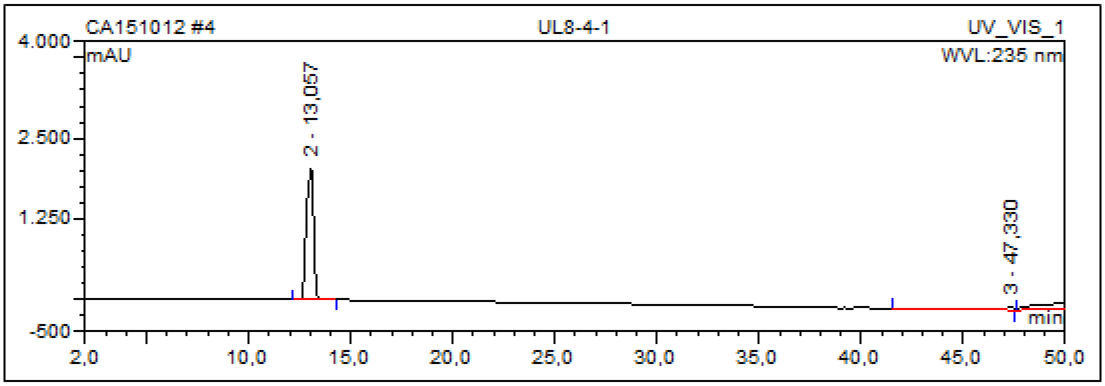

Figure S21. HPLC chromatogram of compound **5**.

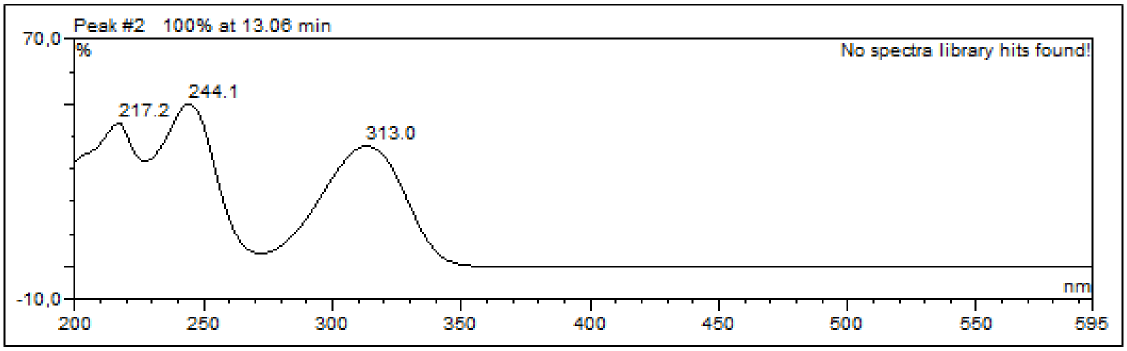

Figure S22. UV spectrum of compound **5**.

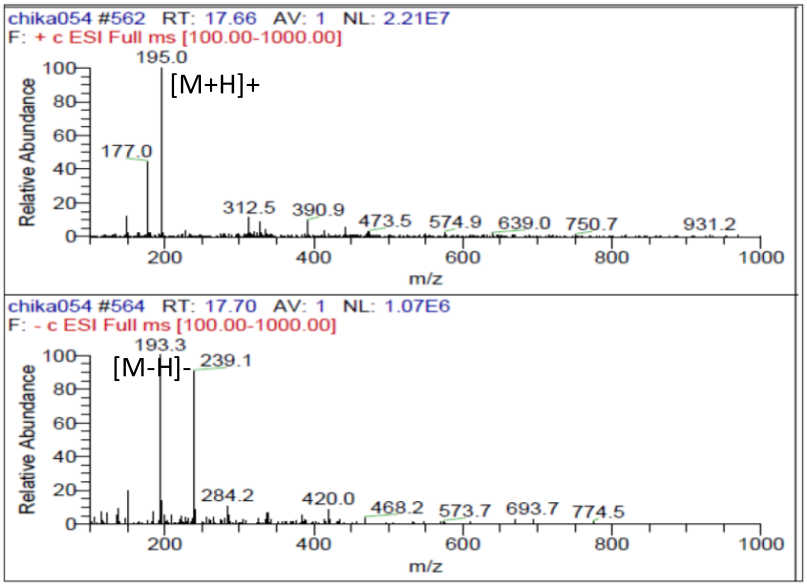

Figure S23. LC-MS of compound **5**.

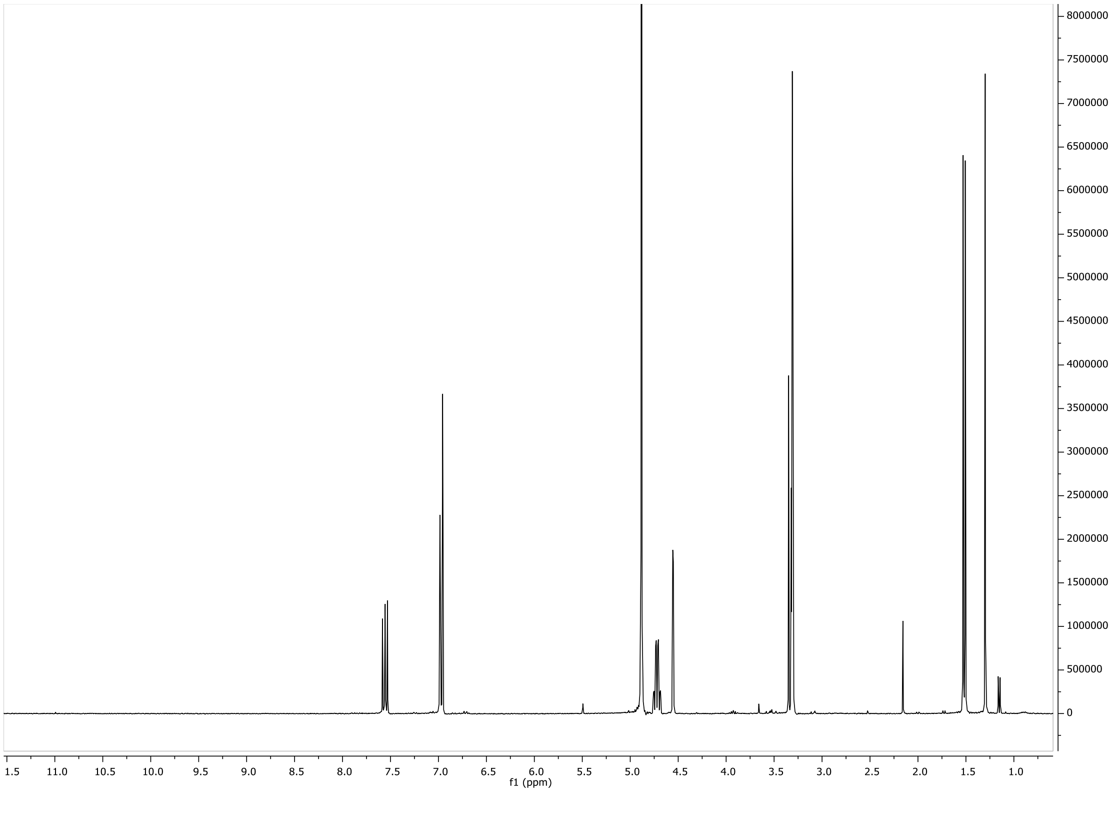

Figure S24. ^{1}H NMR spectrum of **5** at 300 MHz in methanol-d_4.

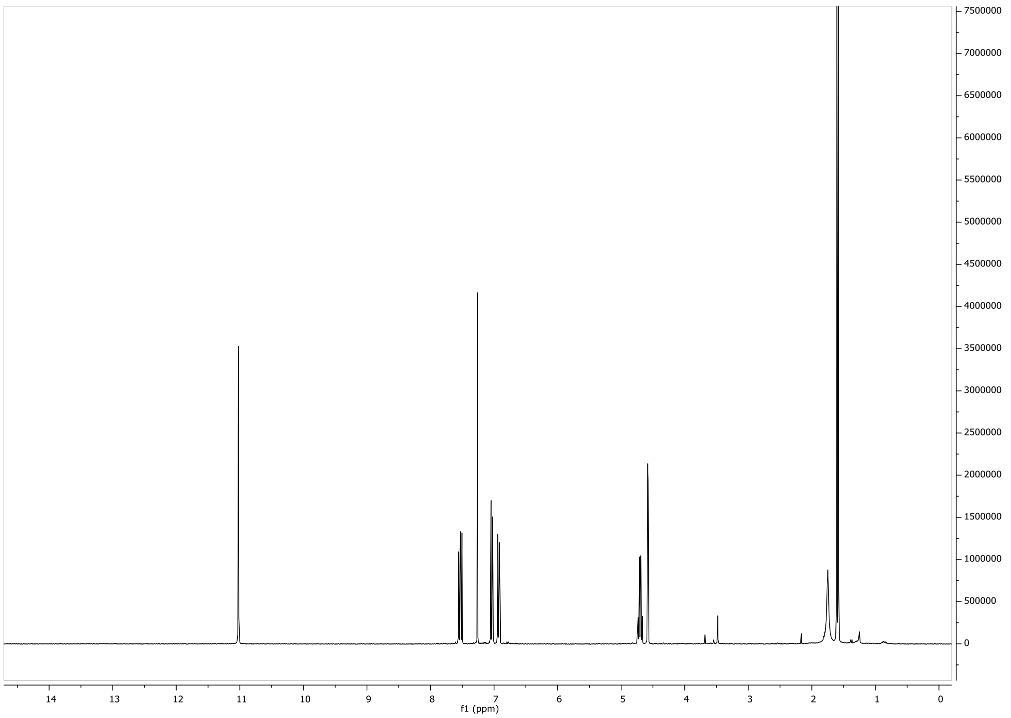

Figure S25. ^{1}H NMR spectrum of **5** at 300 MHz in chloroform-*d*.

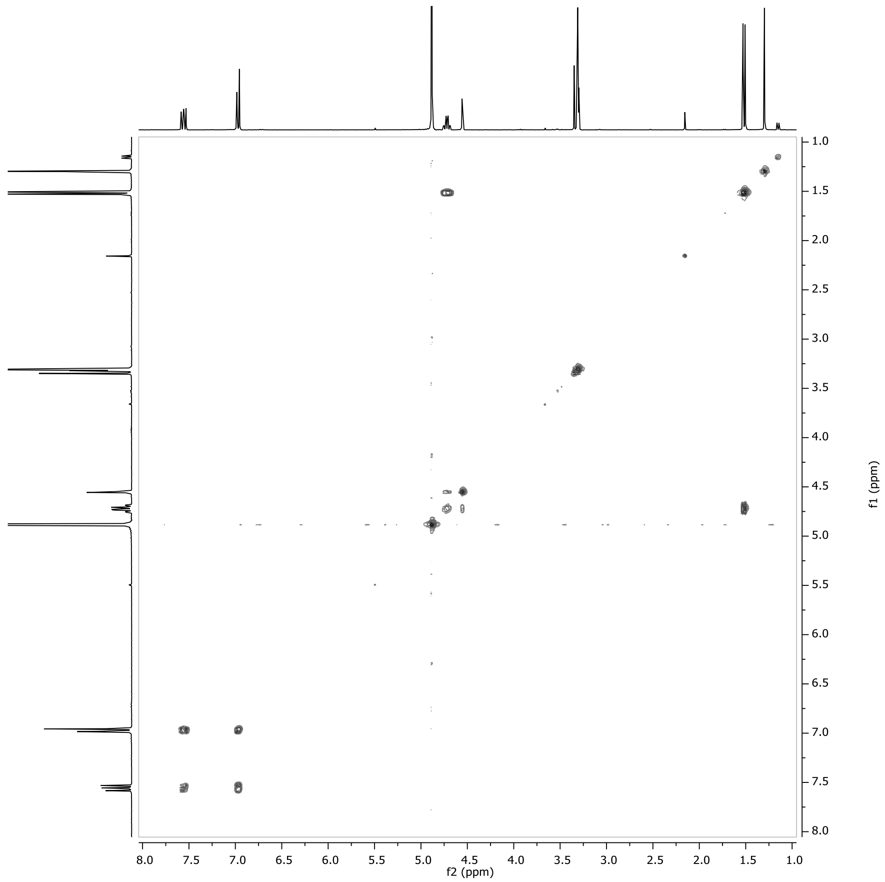

Figure S26. ^{1}H–^{1}H COSY spectrum of **5** at 300 MHz in methanol-d_4.

Table S4. ^{1}H NMR data of **5**.

**Position**	**δ_H^{a} (multi, *J*[Hz])**	**δ_H^{b} (multi, *J*[Hz])**
3	4.72 (qd, 6.6, 2.1)	4.70 (qd, 6.6, 2.1)
4	4.55 (d, 2.1)	4.58 (d, 2.1)
5	6.98 (d, 8.4)	7.03 (dd, 8.5, 1.0)
6	7.56 (dd, 8.4, 7.4)	7.53 (dd, 8.5, 7.3)
7	6.96 (m)	6.93 (dd, 7.3, 1.0)
8-O*H*		11.02 (s)
9	1.52 (d, 6.6, 3H)	1.59 (d, 6.6, 3H)

Measured at 300 MHz in ^{a} methanol-d_4 and ^{b} chloroform-d.

DATA FOR COMPOUND 6

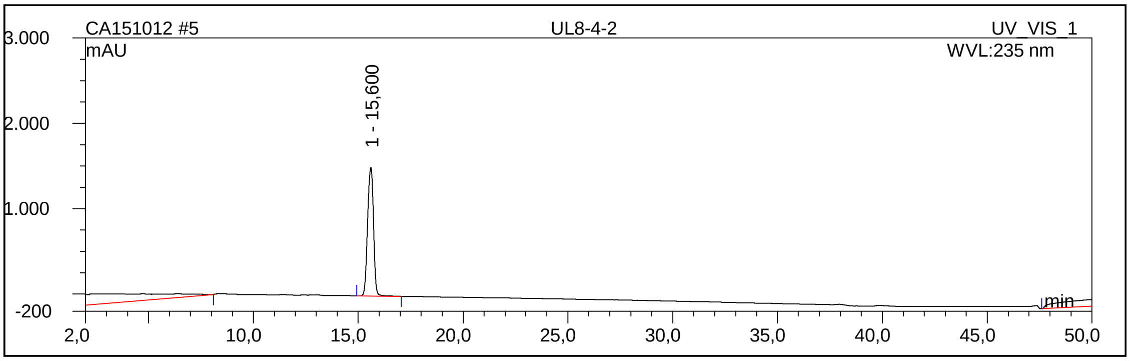

Figure S27. HPLC chromatogram of compound **6**.

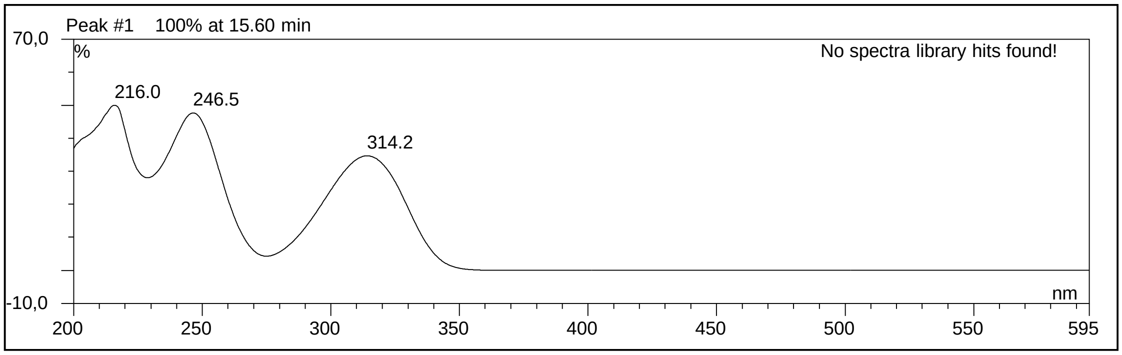

Figure S28. UV spectrum of compound **6**.

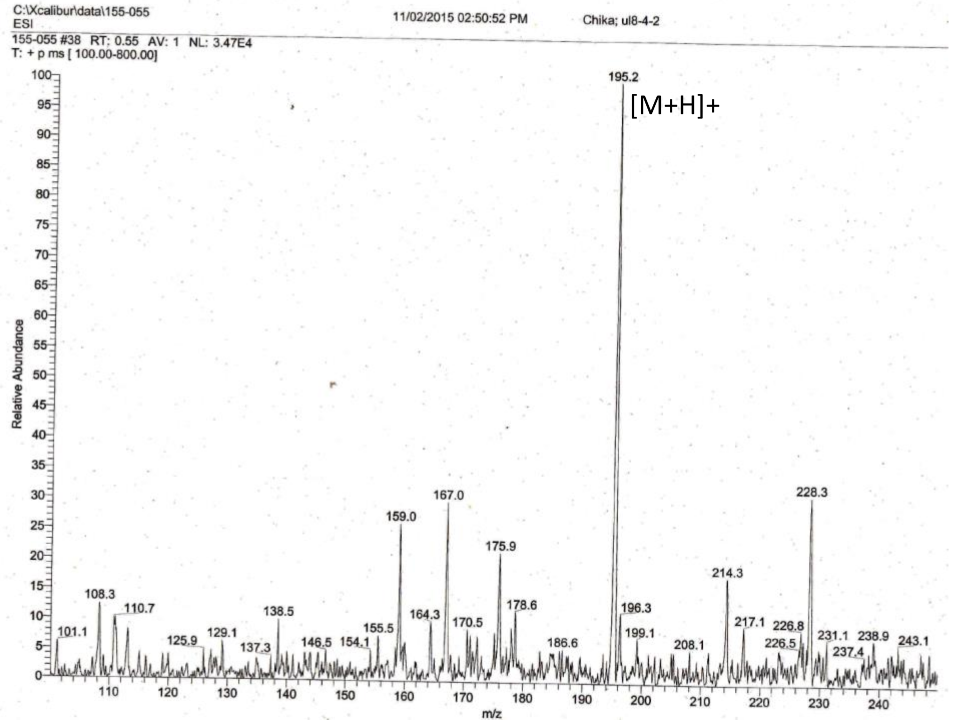

Figure S29. HR-ESI-MS of compound **6**.

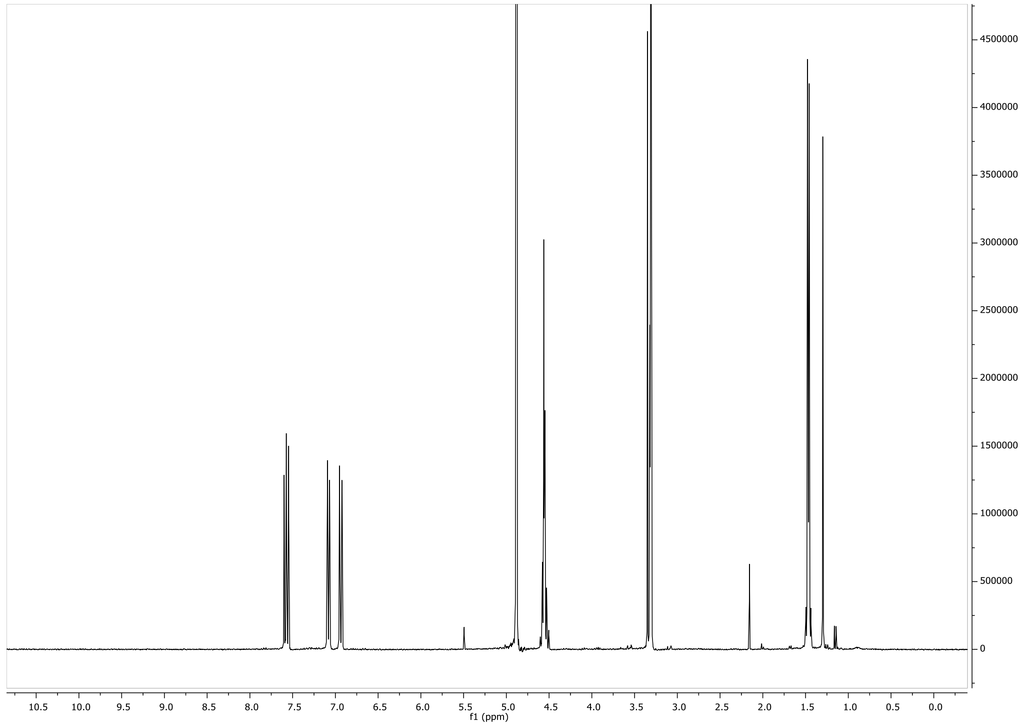

Figure S30. ^{1}H NMR spectrum of **6** at 300 MHz in methanol-d_4.

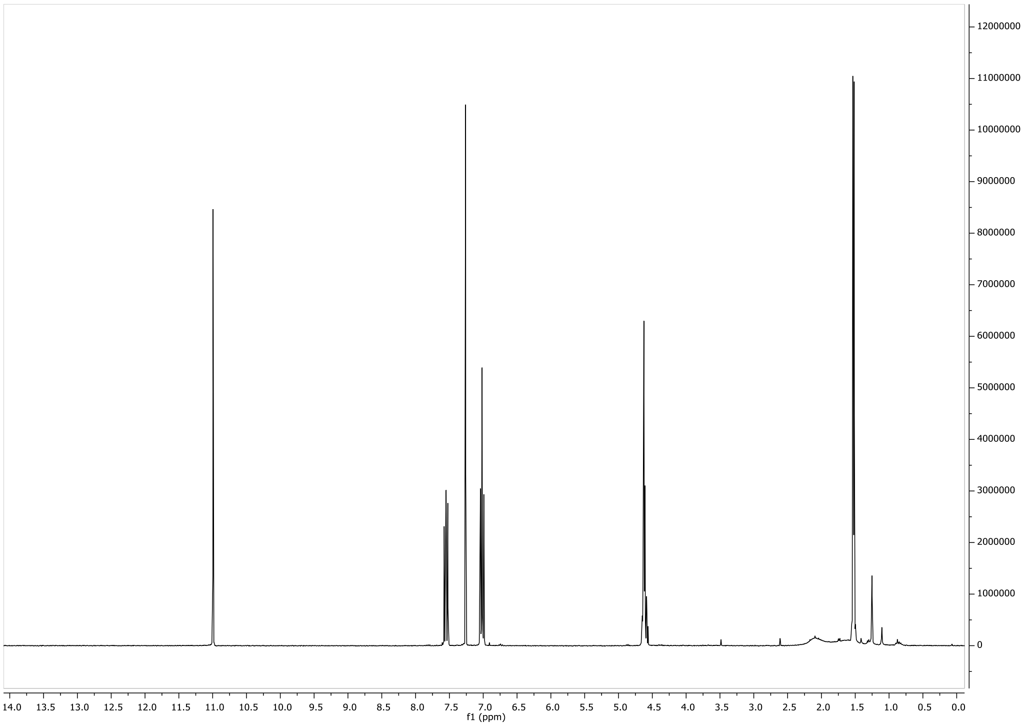

Figure S31. ^{1}H NMR spectrum of **6** at 300 MHz in chloroform-*d*.

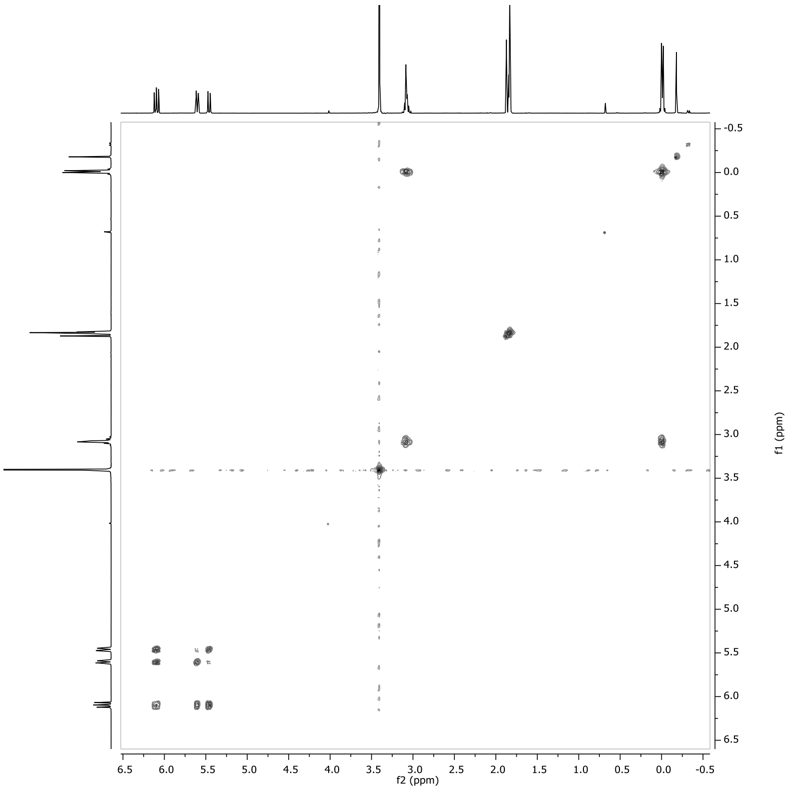

Figure S32. ^{1}H–^{1}H COSY spectrum of **6** at 300 MHz in methanol-*d*$_4$.

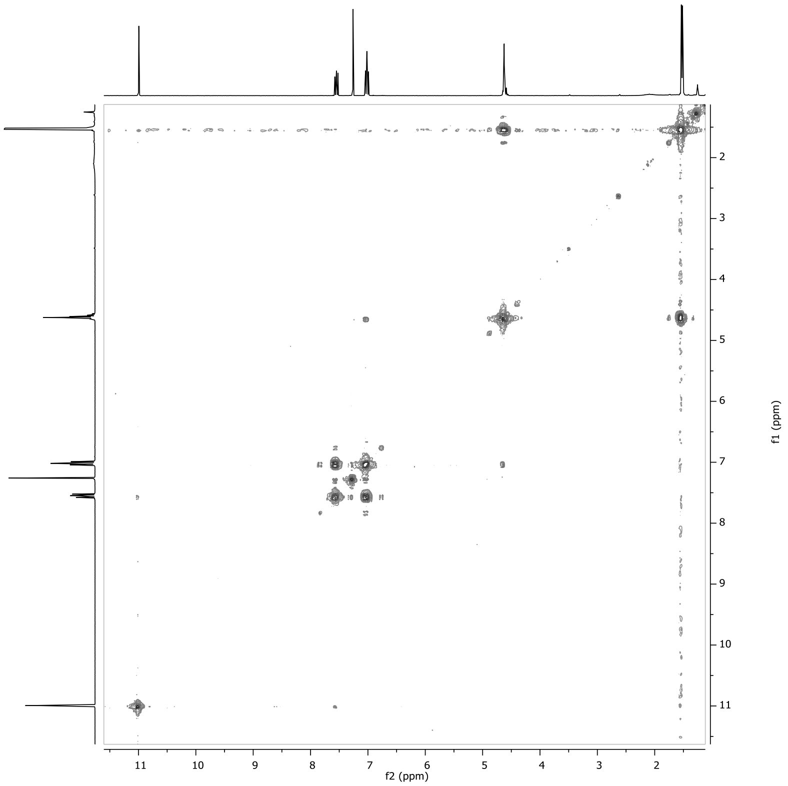

Figure S33. ^{1}H–^{1}H COSY spectrum of **6** at 300 MHz in chloroform-*d*.

Table S5. ^{1}H NMR Data of **6**.

**Position**	**$\delta_H{}^a$ (multi, *J*[Hz])**	**$\delta_H{}^b$ (multi, *J*[Hz])**
3	4.56 (m, overlapped)	4.62 (m, overlapped)
4	4.56 (d, 4.2)	4.62 (d, 5.2)
5	7.08 (dt, 7.3, 0.9)	7.02 (td, 8.0, 1.0)
6	7.57 (dd, 8.4, 7.3)	7.55 (dd, 8.5, 8.5)
7	6.94 (dd, 8.5, 1.1)	7.02 (dd, 8.0, 1.0)
8-O*H*		10.99 (s)
9	1.47 (d, 6.2, 3H)	1.53 (d, 6.2, 3H)

Measured at 300 MHz in [a] methanol-d_4 and [b] chloroform-*d*.
